# Annual Prevalence of Opioid Receipt by South Carolina Medicaid-Enrolled Children and Adolescents: 2000–2020

**DOI:** 10.3390/ijerph20095681

**Published:** 2023-04-28

**Authors:** William T. Basco, David G. Bundy, Sandra S. Garner, Myla Ebeling, Kit N. Simpson

**Affiliations:** 1 Department of Pediatrics, College of Medicine, The Medical University of South Carolina, Charleston, SC 29425, USA; 2 Department of Clinical Pharmacy and Outcome Sciences, College of Pharmacy, The Medical University of South Carolina, Charleston, SC 29425, USA; 3 Department of Healthcare Leadership & Management, College of Health Professions, The Medical University of South Carolina, Charleston, SC 29425, USA

**Keywords:** opioid, children, adolescents, prevalence, Medicaid, race, ethnicity, patient safety

## Abstract

Understanding patterns of opioid receipt by children and adolescents over time and understanding differences between age groups can help identify opportunities for future opioid stewardship. We conducted a retrospective cohort study, using South Carolina Medicaid data for children and adolescents 0–18 years old between 2000–2020, calculating the annual prevalence of opioid receipt for medical diagnoses in ambulatory settings. We examined differences in prevalence by calendar year, race/ethnicity, and by age group. The annual prevalence of opioid receipt for medical diagnoses changed significantly over the years studied, from 187.5 per 1000 in 2000 to 41.9 per 1000 in 2020 (Cochran–Armitage test for trend, *p* < 0.0001). In all calendar years, older ages were associated with greater prevalence of opioid receipt. Adjusted analyses (logistic regression) assessed calendar year differences in opioid receipt, controlling for age group, sex, and race/ethnicity. In the adjusted analyses, calendar year was inversely associated with opioid receipt (aOR 0.927, 95% CI 0.926–0.927). Males and older ages were more likely to receive opioids, while persons of Black race and Hispanic ethnicity had lower odds of receiving opioids. While opioid receipt declined among all age groups during 2000–2020, adolescents 12–18 had persistently higher annual prevalence of opioid receipt when compared to younger age groups.

## 1. Introduction

Although the United States Food and Drug Administration (FDA) has approved childhood and adolescent use of opioid medications for both cough suppression and pain control, multiple professional organizations and the FDA continue to advocate for the reduction of opioid prescription for children and even recommend eliminating use of certain opioids in children and adolescents [1,2,3,4,5,6]. Opioid medications comprise one of the top three drug classes involved in pediatric adverse events leading to emergency department (ED) visits [7,8]. Among pre-adolescent children, two mechanisms of opioid-related adverse events predominate: adverse events experienced when taking the medications as prescribed (e.g., respiratory depression, somnolence, or constipation), and by accidental ingestion of opioid medications prescribed to others or by accidental ingestion of elicit opioids, often from excess opioids found in the home [7,8,9]. Representative data from 40 states in the United States (US) reveals that opioids were involved in 47.3% of fatal poisonings in infants and children up to 5 years old between 2005 and 2018, but the opioids involved represented a mixture of prescription and elicit opioids in homes [9]. The opioid epidemic affects adolescents in different ways than children, with increasing risk of intentional misuse of opioids as children age and additional concerns of diversion of opioids [10,11,12]. Data from a national sample of high school seniors suggest that exposure to doctor-prescribed opioid medications for appropriate medical indications during childhood and adolescence can pre-dispose to opioid misuse in young adulthood [13,14]. Indeed, the past two decades have seen increases in opioid-related hospitalizations nationally among adolescents and a 40% increase in the rate of opioid-related admissions to pediatric intensive care units [15,16]. Data from the Centers for Disease Control and Prevention demonstrate that between 1999 and 2016, the annual child, adolescent, and young adult mortality rate due to exposure to prescription and illicit opioids increased steadily, with a dip between 2008 and 2013, only to climb again, and exceeding the 2008 peak by 2016 [17].

Specific recommendations seeking to reduce opioid prescription to children were made sporadically throughout the 1990s and 2000s, with a seminal recommendation coming in 1997 from the American Academy of Pediatrics (AAP) to avoid codeine for coughs and colds in young children [18]. A subsequent FDA decision requires manufacturers of cough and cold preparations that contain opioids to change the labeling in an attempt to reduce the use of these compounds in persons under 18 [6]. In addition, beginning in 2012 and 2013, there have been specific warnings against using codeine for pain control post-tonsillectomy and adenoidectomy because of the heightened risk of respiratory depression among children who rapidly metabolize codeine to the more potent molecule morphine [3,19,20]. Many states have instituted prescription drug monitoring programs (PDMP) in the past two decades, and robust PDMP requirements on prescribers and pharmacies are associated with declines in opioid prescription among all populations, not just children [21].

There are limited population-based studies describing how ambulatory opioid prescription and dispensing to children and adolescents has changed over long periods of time, especially examining changes among age groups, among races and ethnicities, and including infants and children <2 years old. Existing data suggest that variation in opioid prescription by state and county can be substantial and point to the need to evaluate state-level and regional data [22]. One recent report evaluating Tennessee Medicaid claims that data from 1999 to 2014 demonstrated that, on average, 15% of the subjects received an opioid prescription each year, and the annual prevalence of opioid receipt declined to 10.2% by 2014, having peaked in 2009 [23]. The current study seeks to add to what is known about opioid receipt in ambulatory settings on a population level by utilizing data through 2020, by including subjects 0–2 years old, and by including opioid-containing analgesics along with opioid-containing cough and cold preparations, which comprised 35% of dispensed opioids in previous research using South Carolina (SC) Medicaid data [24]. We sought to evaluate how the annual prevalence of opioid receipt in ambulatory settings has changed over this long time period and also understand changes in prevalence among different age groups. 

## 2. Materials and Methods

### 2.1. Data

We conducted a retrospective cohort study using South Carolina (SC) Medicaid data, obtained as de-identified files from the SC Office of Revenue and Fiscal Affairs (SC ORFA). SC ORFA prepares data sets for investigators by identifying needed files and variables based on queries submitted by prospective investigators. Before releasing the data to investigators, SC ORFA evaluates the data for duplicates, implausible values, and other potentially erroneous entries. This study utilized the “pharmacy” file for prescription claim data (date of prescription dispensing, name of drug, drug National Drug Code (NDC), drug concentration, number of doses, volume dispensed, etc.) for each prescription. We utilized the “recipients” file to obtain the date of birth, sex, race, and ethnicity of each enrollee. Finally, we utilized the “eligibility” file which contains the dates of eligibility for Medicaid. We also used “inpatient” files to identify patients admitted to the hospital. The SC ORFA assigns each subject a unique identifier that is consistent across all files, allowing researchers to merge de-identified data files. In addition, a coded timing variable is provided, measured in days, to allow researchers to know the relative occurrences of each entry in each file. Like the unique identifier, the coded timing variable is consistent across files, so that researchers can tell when a dispensed prescription in the pharmacy file, for example, occurred relative to a visit in the data file containing ambulatory visits. SC ORFA disguises the actual date of any service by choosing a “day zero” among all of the files requested, then timing all requested data relative to that start date. The start date is unknown to researchers.

### 2.2. Subjects

We identified persons 0–18 years old enrolled in SC Medicaid for any portion of a calendar year with at least one pharmacy claim for any drug during that calendar year, as a means of capturing persons who were active in accessing Medicare-funded services and to be able to attribute age at the time of prescription. Analyses were repeated only with subjects who were enrolled continuously for each calendar year (84.7% of those who received at least one prescription), and the findings were similar among continuously enrolled or partial-year enrolled, so the results for all subjects are presented.

### 2.3. Drugs

From the pharmacy data, we identified opioids dispensed to study-eligible persons during each calendar year in the ambulatory setting, excluding opioids dispensed in inpatient settings. The list of opioids was developed from the FDA’s list of controlled substances and further cross-referenced from published studies and known nomenclature conventions (e.g., “HC” in a drug name determined to be hydrocodone) for any additional drug product names [25,26,27,28]. The list of opioids evaluated includes >20,000 opioid-containing NDC codes and is maintained as a database to which new opioid preparations are added when they become available in the US market. We utilized opioids on the US market as of 2020 as our reference list of drugs, but also included opioids that were available in early years of the data but that may have been off the US market by 2020. The list of unique opioid preparations identified among the study sample, in descending frequency, is provided as supplemental material (Supplemental Table). 

### 2.4. Outcomes

The outcome of interest was the annual prevalence of receipt of at least one opioid prescription, as identified in the dispensed pharmacy claims file. Therefore, each enrollee was counted once per calendar year, and each enrollee would be coded as “yes” or “no” for having received an opioid prescription in the respective calendar year. The numerator for prevalence represented those with “yes” values in a calendar year. The denominator for each calendar year was all enrollees in the appropriate age group evaluated who received at least one prescription of any type during that calendar year. Therefore, any person who received a prescription of any type during a calendar year contributed a person-year to that calendar year. All analyses were conducted utilizing SAS, Inc. version 9.4, Cary, N.C. We calculated unadjusted associations between opioid receipt and calendar year using the Cochran–Armitage test for trend. We calculated unadjusted associations of opioid receipt and demographic variables including the age groups, race/ethnicity, and sex (Chi-square analyses) using person-year as the unit of measure. In SC Medicaid data, ethnicity is not delineated separately from the race variable, so we utilized Hispanic ethnicity as equivalent to a racial category in analyses. For adjusted analyses, we utilized logistic regression to assess association with calendar year, adjusting for all demographic variables. This study was deemed “not human research” by the Institutional Review Board at the Medical University of South Carolina. Therefore, informed consent of Medicaid-funded children or their families was not required.

## 3. Results

### 3.1. Descriptive Analyses

**Figure 1:**
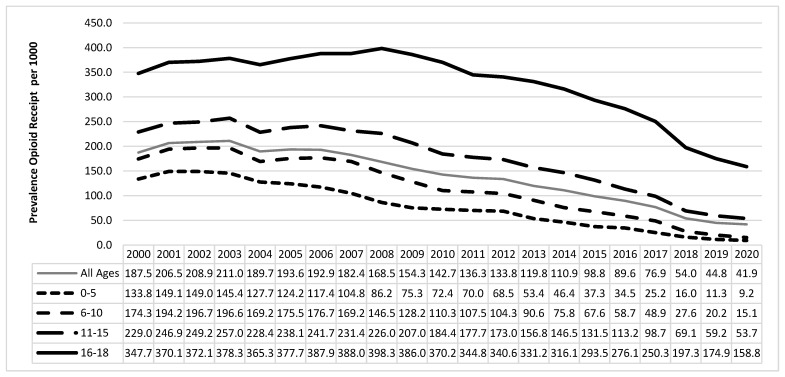
Annual Prevalence^a^ of Opioid Receipt per 1000 Persons, South Carolina Medicaid Enrollees 0–18 years old, by Age Group, 2000–2020. Annual prevalence calculation: numerator = number of persons within age category who received ≥1 opioid in a calendar year; denominator = number of persons who received ≥1 medication of any type in the same calendar year, expressed per 1000 enrollees. An enrollee may appear in multiple years.

**Figure 2:**
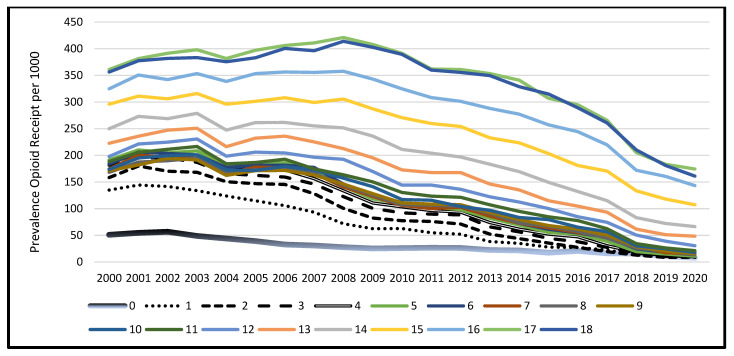
Annual Prevalence^a^ of Opioid Receipt per 1000 Persons, South Carolina Medicaid Enrollees 0–18 years old, by Calendar year and 1-Year Age Strata, 2000–2020. Annual prevalence calculation: numerator = number of persons within age category who received ≥1 opioid in a calendar year; denominator = number of persons who received ≥1 medication of any type in the same calendar year, expressed per 1000 enrollees. An enrollee may appear in multiple years.

There were 2,007,088 unique subjects 0–18 years old who received at least one prescription of any type in at least one year, contributing 8,031,811 person-years of eligibility in the data set. The prevalence of opioid receipt was 135.1 per 1000 Medicaid-funded persons 0–18 years old between 2000–2020. Among all subjects, the annual prevalence of opioid receipt changed during the study years (Figure 1), from 187.5 per 1000 persons per year in 2000 to 41.9 per 1000 persons per year by 2020 (Cochran–Armitage test for trend, *p* < 0.0001). Among all children studied, the prevalence of opioid receipt was greatest in the year 2003, at 211.0/1000/year, and then declined steadily thereafter. When evaluating differences by 1-year age strata (Figure 2), all age groups experienced a reduced annual prevalence of opioid receipt over time (Figure 1 and Figure 2). Older children experienced greater prevalence of opioid receipt regardless of calendar year, and gaps between the 1-year age strata suggest that for each advancing year of age, enrollees were more likely to receive an opioid. 

### 3.2. Unadjusted Analyses

In unadjusted analyses (Table 1), older age groups and female sex were associated with higher prevalence of opioid receipt, while Black race and Hispanic ethnicity were associated with lower prevalence of opioid receipt (Chi-square among groups, all *p* < 0.001). 

### 3.3. Adjusted Analyses

In logistic regression that included age group, race/ethnicity, and sex, calendar year was inversely associated with opioid receipt (Wald Chi-square 573,163.3, *p* < 0.001; Table 2). Black subjects had lower adjusted odds of opioid receipt compared to White subjects (aOR 0.649, 95% CI 0.646, 0.652), as did Hispanic subjects (aOR 0.732; 95% CI 0.723, 0.741). Using White race as the reference, all minority groups had lower adjusted odds of receiving opioids, after controlling for calendar year, age category, and sex. Male sex was associated with greater adjusted odds of opioid receipt in the adjusted model compared to female sex. Despite the annual declines, there were 15,372 children and adolescents 0–18 years old in the data set who received at least one opioid prescription in 2020. 

## 4. Discussion

We found that SC Medicaid-enrolled children and adolescents 0–18 years old experienced a significant downward trend in the annual prevalence of opioid receipt between 2000 and 2020, with data from 2015 to 2020 in particular demonstrating an ongoing and steep decline. These findings are generally encouraging, suggesting that providers are heeding prevalent concerns about excessive opioid prescription and dispensing to children and excessive opioid availability in communities [29,30]. Despite the declines, many persons 0–18 years old continue to receive opioids, and providers often confront the tension of offering needed pain control in complex patients by doing so in a judicious manner [31]. The Joint Commission on Accreditation of Healthcare Organizations mandated that hospitals assess patients for pain in 2001. While this focus on pain assessment was intended for inpatients, hospital-based clinics were also obligated to perform this assessment, and that effort potentially contributed to an unintentional increase in opioid prescription in emergency departments and ambulatory office settings during the 2000s [32,33]. Our data show that the annual prevalence of opioid receipt for children 0–5 years old in SC peaked in 2002 and 2003, perhaps related to the increased national focus on assessing and addressing pain, such as the clinical pain score used by many children’s hospitals [32,33]. The peak years for prevalence of opioid receipt among persons 6–18 years old in this cohort lagged behind the peak years for younger children and occurred in 2007–2008, mirroring other states’ Medicaid data and national data from emergency departments [23,34,35]. 

The factors contributing to the temporal decline in overall opioid prevalence noted in these data are likely multifactorial, including increased awareness about potential dangers of opioid use by the public and providers, along with regulatory actions [26,36,37]. While the changes in the annual prevalence of opioid receipt appeared gradual, some notable milestones occurred during the calendar years studied. First, the AAP’s recommendations discouraging the use of opioids for cough and cold symptoms in young children did not change [18]. During the years of this study, SC and other states instituted PDMP for patients receiving controlled substances as a way for providers to monitor patients and for states to monitor providers and pharmacies, and PDMP programs have been associated with reductions in opioid dispensing for all patients [36,37]. South Carolina’s PDMP was implemented in 2008, with pharmacies required to submit data in 2014, and providers required to check the database when prescribing a controlled substance beginning in 2016. The year 2018 saw implementation of limits on days-supply for opioids, further applying scrutiny and pressure to opioid prescription and dispensing. We suspect that the general scrutiny of all opioid prescription and dispensing happening in South Carolina and across the US and the world contributed to a dampening effect on opioid prescription and dispensing across the age spectrum, demonstrated by the steady declines in opioid dispensing prevalence for all age groups in our study [21,37,38,39]. 

In addition to regulatory efforts, research demonstrating safety concerns about opioids and children published throughout the 2000s and 2010s revealed the downside of prescriber comfort with opioid medications, and entreaties and recommendations from professional organizations such as the American Academy of Pediatrics, along with the Centers for Disease Control and Prevention and the FDA, contributed to public and provider awareness of the risks of adverse events, accidental ingestions, and potential diversion when opioids are prescribed to children and adolescents [20,26,29]. Other notable recommendations in 2012–2013 drew attention to the specific concerns of prescribing codeine after tonsillectomy to children < 2 years old. These entreaties created an environment among pediatric providers that likely led to an extension of provider concern from codeine to all opioids, and from tonsillectomy (with or without adenoidectomy) specifically to other pediatric surgical and non-surgical conditions, further influencing opioid prescription to children [3,5,19,20,40,41]. In 2018, the FDA updated guidance to further limit the prescribing of opioid-containing cough and cold preparations to persons 0–18, likely leading to additional declines beyond the years we evaluated [6]. It is also possible that recent data demonstrating that multi-modal pain treatment approaches using both non-opioid medications and non-medication interventions can be as effective as opioids for managing post-operative or post-traumatic pain has contributed to decreased opioid prescription post-injury or post-operatively [42,43,44].

The findings of age-related differences in the annual prevalence of opioid receipt are consistent with other studies. With increasing age, children and adolescents seen in ED settings or post-surgery are more likely to receive opioids [34,35,45,46,47]. Studies have also consistently demonstrated the association of older ages with adverse drug events, hospitalizations, and death due to opioids [15,17,23,48]. The one-year age-specific data in Figure 2 reveal patterns that suggest opportunities for future opioid stewardship. For example, in earlier calendar years, there were large gaps between the prevalence of opioid receipt curves of infants and 1–2 year old children, and there is a marked jump in annual prevalence of opioid receipt for each advancing year of age beginning at age 12 years (Figure 2). These observations suggest age-related “thresholds” where provider comfort with prescribing opioids becomes more favorable or the clinical indications for which subjects receive opioids shift. While the curves for children aged 0–8 have almost converged, demonstrating that prescribing and dispensing of opioids for these children likely follows similar decision approaches by providers, there remain gaps among each advancing age group, demonstrating that providers’ comfort with and reasons for opioid prescription and dispensing for children and adolescents over 8 years old is different from the approaches to younger children. Further inquiry as to the indications, settings, or provider types for different opioid prescription and dispensing based on these older age thresholds may identify further opportunities to improve opioid stewardship for older children and adolescents.

The finding of lower prevalence of opioid receipt by African American and Hispanic persons echoes findings of other studies evaluating analgesic treatment of racial and ethnic minorities. Data from the National Hospital Ambulatory Medical Care Survey from 2006–2015 demonstrate that White children and adolescents were more likely to receive opioids at ED visits, for any cause, than were non-White children and adolescents [34]. Data from the Medical Expenditure Panel Survey from 2003–2014 similarly demonstrated that non-Hispanic Black, Hispanic, and Asian children and adolescents were all less likely to receive opioids compared to White children and adolescents, with similar findings from regional and local studies [49,50,51,52]. The drivers of decreased receipt of opioids by minority children and adolescents are likely multifactorial, but they may include patient cultural differences in concerns about opioids as well as provider bias in management of pain and the perceived risks of prescribing opioids to persons of non-White racial or Hispanic backgrounds [53,54,55,56]. An ongoing focus on racial and ethnic differences in the receipt of opioids for both indicated conditions (e.g., after injury) and for non-indicated conditions (e.g., respiratory illnesses) should remain an important part of opioid stewardship research as the data demonstrating the racial differences in opioid receipt demonstrate persistent differences even among more recent calendar years [49,50,51,52].

While the findings of this and other research evaluating child opioid use demonstrate encouraging decreases in opioid receipt by children, thoughtful, consistent opioid stewardship is essential to the safe use of these medications. Ongoing attempts to reduce opioid prescription for non-indicated illnesses, such as headache or respiratory illnesses, will be important aspects of opioid stewardship efforts [57,58,59]. In addition, studies have repeatedly demonstrated that pediatric surgical patients are dispensed more opioids than they use post-operatively, with patients generally taking < 50% of the prescribed doses after discharge from a procedure [60,61,62,63]. Many patients consciously decide to keep unused pain medication for future use, signifying a need to further educate parents on the risk of unused opioids in the household and the need for proper disposal [64]. Unused opioids retained in the household are often the source of accidental child opioid ingestion [7,8,9]. Therefore, future studies on proper prescription by indication and prescribing appropriate days-supply and doses are important to conduct, despite decreases in overall opioid prescription, dispensing, and receipt.

There are several limitations to our study. First, the data represent only one state, and there is considerable variability in opioid prescription among individual states in the US [22,65]. These data represent the experiences of only Medicaid-insured patients, but those patients represent approximately 60% of the children born in South Carolina. Nonetheless, the trends we note can only be potentially extrapolated to other publicly funded populations and not children who are privately-insured or uninsured. There are inherent limitations in using pharmacy claims data. For example, we do not know the frequency of opioid prescriptions written but not dispensed, or dispensed but paid for by non-Medicaid methods (e.g., cash purchases), both of which result in underestimates of the annual opioid prevalence among children and adolescents in these data. Claims data also do not capture patient use of the opioids once they have been prescribed and dispensed. Because of those two limitations, we have generally used the term “dispensing” to refer to the events measured in this study rather than terms such as “prescribing” or “use,” but all three terms represent separate and important aspects of studying opioid exposure in children and adolescents and have policy implications. Claims data do not provide important information about the indications for which a drug is prescribed, and future opioid stewardship efforts would be greatly aided by requiring providers to list indications when prescribing these medications. We also do not know how many of the drugs were prescribed to be taken on a scheduled versus an “as needed” basis, making it difficult to assess true exposure to opioids by the children and adolescents studied. Provider type and treatment setting, missing from the pharmacy data we obtained, are also important to consider when devising stewardship efforts, as other studies have demonstrated that provider type (e.g., “non-pediatrician”) and location (e.g., emergency department versus ambulatory office settings) are associated with different likelihood of patient receipt of opioids [58,60]. This study also did not account for repeat opioid prescriptions per person, but instead used a per person per year approach; therefore these data under-estimate the total opioid exposure in each calendar year. The denominator is limited to only persons who received at least one prescription in a calendar year, chosen to identify subjects who were truly active in the Medicaid program and as a proxy for medical access. Our post hoc evaluation revealed that >80% of enrollees received at least one dispensed prescription for a drug in any given year. While the point estimates of rates would have changed using a different denominator, such as all Medicaid enrollees in each calendar year, we do not believe that the main conclusions would have changed substantially.

In November 2022, the CDC released updated guidelines for prescribing opioids for pain [66]. These guidelines were intended to apply only to adult patients ≥ 18 years old, but the suggestions hold value for the treatment of pain in children and adolescents as well. Selected recommendations that would apply well to children and adolescent patients include: Providers should focus on non-opioid medications and non-medication treatment options for pain, focusing on multi-modal approaches; should prescribe immediate-release opioids for acute pain rather than long-acting or extended release opioids when opioids are used; should strive for the lowest effective dose and shortest duration of opioid treatment when opioid treatment is needed; should avoid co-prescribing of opioids with other medications that can raise the risk of adverse events, such as benzodiazepines; and should make sure parents and adolescents understand the risks of opioid use [66].

## 5. Conclusions

The calendar year 2020 annual prevalence of opioid receipt by Medicaid-funded persons 0–18 years old in South Carolina was 22.3% of the calendar year 2000 annual prevalence, but older children and adolescents still experience a substantial annual prevalence of opioid receipt. Overall, children and adolescents who were members of minority racial or ethnic groups were less likely to receive opioids compared to White children and adolescents after adjusting for calendar year and sex. Male children and adolescents were more likely to receive opioids compared to female children and adolescents in the fully adjusted model. While the overall decrease in prevalence of opioid receipt is encouraging, further inquiry is needed to evaluate the appropriateness of current prescriptions dispensed for the thousands of children and adolescents still receiving opioids.

## Figures and Tables

**Table 1:** Unadjusted Associations with Receipt of at Least 1 Opioid Prescription, South Carolina Medicaid Children 0–18 years old, 2000–2020 ^a^.

Variable	Total N (Person-Years)	Received Opioid N Prevalence (95% CI) ^c^	No Opioid N Prevalence (95% CI) ^c^
**Age group (years) ^b^**			
**0–5**	3,261,659	250,218 76.7 (76.4, 77.0)	3,011,441 923.3 (922.2, 924.3)
**6–10**	2,043,264	219,082 107.2 (106.8, 107.7)	1,824,182 892.8 (891.5, 894.1)
**11–15**	1,698,041	285,284 168.0 (167.4, 168.6)	1,412,757 832.0 (830.6, 833.4)
**16–18**	1,028,847	330,186 320.9 (319.8, 322.0)	698,661 679.1 (677.5, 680.7)
**Sex** ^b^			
**Female**	4,003,077	563,812 140.8 (140.5, 141.2)	3,439,265 859.2 (858.2, 860.1)
**Male**	4,028,482	520,900 129.3 (129.0, 129.7)	3,507,582 870.7 (869.8, 871.6)
**Race/Ethnicity** ^**b,d**^			
**White**	3,046,257	514,325 168.8 (168.4, 169.3)	2,531,932 831.2 (830.1, 832.2)
**Black**	3,376,191	428,202 126.8 (126.5, 127.2)	2,947,989 873.2 (872.2, 874.2)
**Hispanic**	318,014	32,937 103.6 (102.5, 104.7)	285,077 896.4 (893.1, 899.7)
**Other**	1,291,349	109,306 84.6 (84.1, 85.1)	1,182,043 915.4 (913.7, 917.0)

^a^. Chi-square comparisons among all variables in Table 1 is significant at *p* < 0.0001. ^b^. Chi-square comparisons among groups within each variable (e.g among race/ethnicity groups) are all significant at <0.001. ^c^. Annual prevalence calculation: numerator = number of persons who received ≥1 opioid in a calendar year; denominator = number of persons who received ≥1 medication of any type in the same calendar year, expressed per 1000 enrollees. An enrollee may appear in multiple years. ^d^. SC Medicaid data records “Hispanic” ethnicity as a racial category rather than an ethnic modification of any race.

**Table 2:** Adjusted Model Predicting Receipt of at Least 1 Opioid Prescription in any Calendar Year by South Carolina Medicaid Children 0–18 years old, 2000–2020.

Variable		Adjusted ^a^ Odds Ratio (95% Confidence Intervals)
**Calendar Year**		0.927 (0.926, 0.927)
**Age Category**	0–5 years	reference
	6–10 years	1.525 (1.515, 1.534)
	11–15 years	2.580 (2.565, 2.595)
	16–18 years	6.284 (6.246, 6.322)
**Race/Ethnicity**	White race	reference
	Black race	0.649 (0.646, 0.652)
	Hispanic ethnicity ^b^	0.732 (0.723, 0.741)
	Other race	0.736 (0.731, 0.741)
**Sex**	Female	reference
	Male	1.016 (1.011, 1.020)

^a^. Wald Chi-square 573163.3, *p* < 0.001. Logistic regression adjusts for all variables listed in Table 2. ^b^. SC Medicaid data records “Hispanic” ethnicity as a racial category rather than an ethnic modification of any race.

## Data Availability

The South Carolina Office of Revenue and Fiscal Affairs makes SC Medicaid data available to investigators in de-identified format via request, but investigators are not allowed to share the data with other investigators.

## Supplementary Materials

The following supporting information can be downloaded at: https://www.mdpi.com/article/10.3390/ijerph20095681/s1, Supplementary Data—List of Opioid Drug Preparations studied in South Carolina Medicaid Pharmacy Claims, 2000–2020, Prescribed to Persons 0–18 Years Old.
