# Supplementary material for: Annual Prevalence of Opioid Receipt by South Carolina Medicaid-Enrolled Children and Adolescents: 2000–2020
Source: Int J Environ Res Public Health. 2023 Apr 28;20(9):5681. doi: 10.3390/ijerph20095681 (PMC10178489; doi:10.3390/ijerph20095681)
Supplement: Supplementary file 1 [file ijerph-20-05681-s001.zip › ijerph-2313368-supplementary.pdf]

Supplementary Data—List of Opioid Drug Preparations studied in South Carolina Medicaid Pharmacy Claims, 2000–2020, Prescribed to Persons 0-18 Years Old

DRUG NAME	Frequency	Percent	Cumulative Frequency	Cumulative Percent
ACETAMINOPHEN/COD ELIXIR	87588	8.07	87588	8.07
HYDROCODONE-ACETAMINOPHEN 7.5	86465	7.97	174053	16.05
HYDROCODONE-ACETAMINOPHEN 5 M	79794	7.36	253847	23.40
ACETAMINOPHEN-CODEINE 120-12M	75863	6.99	329710	30.39
ACETAMINOPHEN/COD #3 TABLET	46233	4.26	375943	34.66
HYDROCODONE/APAP 5/500 TAB	43535	4.01	419478	38.67
ACETAMINOPHEN-CODEINE 300MG-3	40125	3.70	459603	42.37
OXYCODONE-ACETAMINOPHEN 5 MG-	35488	3.27	495091	45.64
TRAMADOL HCL 50 MG TABLET	32222	2.97	527313	48.61
ACETAMINOPHEN-CODEINE ELIXIR	25802	2.38	553115	50.99
PROPOXY-N/APAP 100-650 TAB	24350	2.24	577465	53.23
HYDROCODONE-APAP SOLUTION	22244	2.05	599709	55.28
CHERATUSSIN AC 10-100MG/5 LIQ	21609	1.99	621318	57.28
HYDROCODONE-APAP 5-500 TABLET	17969	1.66	639287	58.93
HYDROCODONE W/APAP ELIXIR	15886	1.46	655172	60.40
ACETAMINOPHEN-COD #3 TABLET	15268	1.41	670440	61.80
HISTINEX HC SYRUP	12750	1.18	683190	62.98
PROMETHAZINE/CODEINE SYRUP	12381	1.14	695571	64.12
HYDROCODONE-ACETAMINOPHEN 10M	12111	1.12	707682	65.24
HYDROCODONE-HOMATROPINE MBR 5	11037	1.02	718719	66.26
OXYCODONE W/APAP 5/325 TAB	10461	0.96	729180	67.22
TUSSIONEX PENNKINETIC SUSP	10385	0.96	739564	68.18
HYDROCODONE/APAP 7.5/500 TB	9991	0.92	749555	69.10
HYDROCODON-ACETAMINOPHEN 5-50	9865	0.91	759420	70.01
OXYCODONE W/APAP 5/500 CAP	8284	0.76	767704	70.77
HISTINEX PV SYRUP	7984	0.74	775688	71.51
HYDROCODONE-ACETAMINOPHEN SOL	7865	0.73	783553	72.23
OXYCODONE HCL 5 MG TABLET	7032	0.65	790585	72.88
PROMETHAZINE-CODEINE 6.25-10/	6536	0.60	797121	73.48
HYDROMET 5-1.5 MG/5 SYRUP	6261	0.58	803382	74.06
DE-CHLOR HC LIQUID	6051	0.56	809433	74.62
CHERATUSSIN AC SYRUP	5304	0.49	814737	75.11
OXYCODONE-ACETAMINOPHEN 5-325	5220	0.48	819957	75.59
HYDROCODON-ACETAMINOPHEN 5-32	5056	0.47	825013	76.05
HYDROCODONE-APAP 7.5-500 MG/1	5034	0.46	830047	76.52
ANAPLEX HD LIQUID	4900	0.45	834947	76.97
HYDROCODONE/GUAIFENESIN SYR	4850	0.45	839797	77.42
PROPOXYPHENE NAP-ACETAMINOPHE	4727	0.44	844524	77.85

HYDROCODONE-APAP 7.5-500 TAB	4306	0.40	848830	78.25
HYDROCODONE COMPOUND SYRUP	4281	0.39	853111	78.64
ACETAMINOPHEN-CODEINE SOLUTIO	4280	0.39	857391	79.04
H-C TUSSIVE SYRUP	4271	0.39	861662	79.43
ATUSS HD LIQUID	4212	0.39	865874	79.82
HYDROCODONE/APAP 7.5/750 TB	3854	0.36	869728	80.18
OXYCODONE HCL 5 MG/5 ML SOLUT	3850	0.35	873578	80.53
GUIATUSS AC SYRUP	3692	0.34	877270	80.87
HYPHED SYRUP	3644	0.34	880914	81.21
HYDROCODONE/APAP 10/500 TAB	3554	0.33	884468	81.54
OXYCODONE-ACETAMINOPHEN 10MG-	3522	0.32	887990	81.86
MYTUSSIN DAC SYRUP	3480	0.32	891470	82.18
OXYCODONE-APAP 5-325 MG TAB	3306	0.30	894776	82.49
PROMETHAZINE VC/COD SYRUP	3269	0.30	898045	82.79
HYDROMET SYRUP	3242	0.30	901287	83.09
ATUSS G SYRUP	3209	0.30	904496	83.38
GUIATUSS DAC SYRUP	3067	0.28	907563	83.66
PROPOXYPHEN-APAP 100-650 MG T	3008	0.28	910571	83.94
HYDROCOD/PHENYLE/CP SYRUP	2787	0.26	913358	84.20
ULTRACET TABLET	2761	0.25	916119	84.45
MYTUSSIN AC SYRUP	2741	0.25	918860	84.71
HYDROCODON-ACETAMIN 7.5-325/1	2606	0.24	921466	84.95
CHERATUSSIN DAC SYRUP	2561	0.24	924027	85.18
CYTUSS HC SYRUP	2454	0.23	926481	85.41
HYDROCODON-ACETAMINOPH 7.5-50	2419	0.22	928900	85.63
ATUSS HC SYRUP	2379	0.22	931279	85.85
GUAIFENESIN W/CODEINE LIQ	2227	0.21	933506	86.06
ATUSS MS LIQUID	2215	0.20	935721	86.26
VICOPROFEN 200/7.5 TABLET	2212	0.20	937933	86.46
HYDRO PC II SYRUP	2142	0.20	940075	86.66
ACETAMINOPHEN/COD #2 TABLET	1985	0.18	942060	86.84
Q-V TUSSIN SYRUP	1944	0.18	944004	87.02
B-TUSS LIQUID	1919	0.18	945923	87.20
HYDRO-DP SYRUP	1890	0.17	947813	87.37
ANAPLEX-HD LIQUID	1850	0.17	949663	87.55
HISTEX HC LIQUID	1842	0.17	951505	87.72
HYDROCODONE-APAP 10-500 TABLE	1827	0.17	953332	87.88
HYDRON CP LIQUID	1824	0.17	955156	88.05
COUGHTUSS LIQUID	1796	0.17	956952	88.22
BROMPLEX HD LIQUID	1676	0.15	958628	88.37
PANCOF HC LIQUID	1577	0.15	960205	88.52
CODEINE-GUAIFENESIN 10-100MG/	1535	0.14	961740	88.66
OXYCODONE-ACETAMINOPHEN 7.5-3	1487	0.14	963227	88.80
HYDROCODONE/APAP 10/650 TAB	1472	0.14	964699	88.93
ULTRAM 50MG TABLET	1454	0.13	966153	89.07
ENDAL-HD SYRUP	1450	0.13	967603	89.20
HYDROCODONE/APAP 7.5/650 TB	1442	0.13	969045	89.33
GUAIFENESIN AC 10-100MG/5 LIQ	1432	0.13	970477	89.46
HYDROCODONE BT-IBUPROFEN TB	1415	0.13	971892	89.59
IOPHEN-C NR 10-100MG/5 LIQUID	1405	0.13	973297	89.72

ACETAMINOP-CODEINE 120-12 MG/	1396	0.13	974693	89.85
HYDROCODONE-ACETAMN 7.5-325/1	1383	0.13	976076	89.98
HYDROCODON-ACETAMINOPH 7.5-32	1346	0.12	977422	90.10
HYDROCODONE-APAP 7.5-750 TAB	1343	0.12	978765	90.23
ROXICET 5/325 TABLET	1340	0.12	980105	90.35
HYDROCODONE-ACETAMIN 5-325 MG	1339	0.12	981444	90.48
VI-Q-TUSS SYRUP	1260	0.12	982704	90.59
CAPITAL W/CODEINE ORAL SUSP	1258	0.12	983962	90.71
GUAIATUSSIN AC 10-100MG/5 LIQ	1234	0.11	985196	90.82
GUAIFENESIN-CODEINE 10-100MG/	1214	0.11	986410	90.93
TRAMADOL HCL 50MG TABLET	1175	0.11	987585	91.04
SU-TUSS HD ELIXIR	1174	0.11	988759	91.15
LEVALL 5.0 SYRUP	1164	0.11	989923	91.26
TRAMADOL HCL-ACETAMINOPHEN TA	1134	0.10	991057	91.36
M-END SYRUP	1114	0.10	992171	91.46
TRAMADOL HCL-ACETAMINOPHEN 37	1104	0.10	993275	91.57
OXYCODONE-APAP 5-500 MG CAP	1066	0.10	994341	91.66
HYDRO-TUSSIN HC LIQUID	1063	0.10	995404	91.76
HYDROCODONE/APAP 2.5/500 TB	995	0.09	996399	91.85
GANI-TUSS-DM NR LIQUID	993	0.09	997392	91.95
OXYCODONE HCL 5 MG/5 ML SOL	962	0.09	998354	92.03
OXYCODONE HCL 5 MG/5 ML SOLN	952	0.09	999306	92.12
HYDROCODON-ACETAMINOPHN 10-50	928	0.09	1000234	92.21
HISTINEX HC 5-2.5-2 SYRUP	922	0.08	1001156	92.29
HYDRON EX LIQUID	922	0.08	1002078	92.38
ATUSS EX SYRUP	918	0.08	1002996	92.46
PROMETHAZINE W/COD SYRUP	913	0.08	1003909	92.55
TRI-VENT HC SYRUP	901	0.08	1004810	92.63
HYDROCODON-ACETAMIN 7.5-500/1	893	0.08	1005703	92.71
MEPERIDINE 50 MG TABLET	890	0.08	1006593	92.79
ENDOCET 5/325 TABLET	876	0.08	1007469	92.87
TUSSIONEX 10-8MG/5ML SUS ER 1	849	0.08	1008318	92.95
PROMETHAZINE VC-CODEINE 6.25-	844	0.08	1009162	93.03
CHERATUSSIN DAC 30-10-100 SYR	840	0.08	1010002	93.11
PHENYLEPH/HCOD BT/CP LIQUID	840	0.08	1010842	93.19
MINTUSS G SYRUP	837	0.08	1011679	93.26
ATUSS MS SYRUP	823	0.08	1012502	93.34
BROVEX HC LIQUID	802	0.07	1013304	93.41
NALEX DH LIQUID	797	0.07	1014101	93.49
HYDROCODONE-IBUPROFEN 7.5-200	782	0.07	1014883	93.56
PNEUMOTUSSIN 2.5 SYRUP	775	0.07	1015658	93.63
MEPERIDINE HCL 50 MG TABLET	774	0.07	1016432	93.70
ENDACOF-HC LIQUID	771	0.07	1017203	93.77
PANCOF XP LIQUID	749	0.07	1017952	93.84
DONATUSSIN DC SYRUP	748	0.07	1018700	93.91
OXYCODONE/APAP 10/650 TAB	739	0.07	1019439	93.98
PROMETHAZINE-CODEINE SYRUP	729	0.07	1020168	94.04
DE-CHLOR HC 10-2.5-2/5 SYRUP	727	0.07	1020895	94.11
HYDROCODON-ACETAMINOPHN 10-32	719	0.07	1021614	94.18
IOPHEN-C NR LIQUID	715	0.07	1022329	94.24

ANAPLEX HD 30-1.7-2/5 SOLUTIO	702	0.06	1023031	94.31
HYDROCODONE-ACETAMINOPHEN 2.5	699	0.06	1023730	94.37
PHENYLEPH/HCOD BT/CP SYRUP	696	0.06	1024426	94.44
HYDRO-PC LIQUID	691	0.06	1025117	94.50
HYDROCODONE-HOMATROPINE SYRUP	684	0.06	1025801	94.56
MEPERIDINE 50MG/5ML SYRUP	662	0.06	1026463	94.63
TRIACIN C COUGH SYRUP	652	0.06	1027115	94.69
PHENYLHISTINE EXPECTORANT	646	0.06	1027761	94.74
MINTUSS HC SYRUP	620	0.06	1028381	94.80
HYDRO-TUSSIN DHC SYRUP	600	0.06	1028981	94.86
ACETAMINOPHEN-CODEINE 300MG-1	586	0.05	1029567	94.91
ENDOCET 5 MG-325MG TABLET	571	0.05	1030138	94.96
HYDROCODONE-APAP 7.5-650 TAB	568	0.05	1030706	95.02
MEPERIDINE 50MG TABLET	567	0.05	1031273	95.07
MEPERIDINE 50 MG/5 ML SOLUTIO	559	0.05	1031832	95.12
OXYCODONE-ACETAMINOPHEN 10-32	552	0.05	1032384	95.17
GANI-TUSS NR LIQUID	550	0.05	1032934	95.22
HISTUSSIN HC SYRUP	528	0.05	1033462	95.27
MEPERIDINE HCL 50 MG/5 ML SOL	526	0.05	1033988	95.32
HYDRO-TUSSIN HD SOLUTION	511	0.05	1034499	95.37
HYDROCODONE-APAP 10-650 TABLE	511	0.05	1035010	95.41
HYDROCODONE-APAP 5-325 TABLET	497	0.05	1035507	95.46
HYDROCODONE-CHLORPHENIRAMNE E	484	0.04	1035991	95.50
PAREGORIC LIQUID	479	0.04	1036470	95.55
DURADAL HD SYRUP	443	0.04	1036913	95.59
HYDROCODON-ACETAMINOPH 7.5-75	443	0.04	1037356	95.63
CYNDAL HD SYRUP	437	0.04	1037793	95.67
PROLEX DH LIQUID	435	0.04	1038228	95.71
DRITUSS HD ELIXIR	434	0.04	1038662	95.75
ENDACOF-XP SYRUP	426	0.04	1039088	95.79
HYDROCODONE/APAP 10/325 TAB	425	0.04	1039513	95.83
HYDROCODONE-APAP 5/500 TAB	422	0.04	1039935	95.87
TRIPROL/P-EPHED/CODEINE SYR	417	0.04	1040352	95.91
MYTUSSIN DAC 30-10-100 SYRUP	416	0.04	1040768	95.94
HYDROCODONE-ACETAMIN 7.5-325	406	0.04	1041174	95.98
HYCET SOLUTION	392	0.04	1041566	96.02
ROXILOX 500/5 CAPSULE	391	0.04	1041957	96.05
HYPHED 30-2.5-2/5 SYRUP	385	0.04	1042342	96.09
OXYCODONE-ACETAMINOPHEN 5-500	378	0.03	1042720	96.12
PROTUSS SOLUTION	377	0.03	1043097	96.16
HISTINEX PV 30-2.5-2/5 SYRUP	376	0.03	1043473	96.19
BROMPHENIRAMINE-HYDROC-PSE LI	374	0.03	1043847	96.23
P-EPD HCL/HCOD BT/CARBIN LQ	370	0.03	1044217	96.26
HYDROCOD BIT-PHENYLEPHRINE-CP	369	0.03	1044586	96.30
HALOTUSSIN-AC LIQUID	355	0.03	1044941	96.33
HYDRO-TUSSIN HD LIQUID	355	0.03	1045296	96.36

HYDROCODONE-GUAIFENESIN 100-5	350	0.03	1045646	96.39
HYDROCODONE-APAP 2.5-500 TAB	345	0.03	1045991	96.43
ENDAL HD SYRUP	343	0.03	1046334	96.46
BROMCOMP HC LIQUID	341	0.03	1046675	96.49
MINTUSS MS SYRUP	339	0.03	1047014	96.52
GUAIFENESIN W/CODEINE 100-10M	331	0.03	1047345	96.55
ROXICODONE 5MG TABLET	324	0.03	1047669	96.58
HYDROCODONE/HOMATROPINE TAB	323	0.03	1047992	96.61
METHADONE 5 MG/5 ML SOLUTION	322	0.03	1048314	96.64
ATUSS HS SUSPENSION	315	0.03	1048629	96.67
ENDOCET 10/650 MG TABLET	307	0.03	1048936	96.70
MYTUSSIN AC 100-10MG/5 LIQUID	299	0.03	1049235	96.72
CRANTEX HC SYRUP	298	0.03	1049533	96.75
BROMPLEX HD 30-1.7-2/5 SOLUTI	297	0.03	1049830	96.78
OXYCODONE-APAP 10-325 MG TAB	289	0.03	1050119	96.81
ENDOCET 5-325 TABLET	288	0.03	1050407	96.83
TRAMADOL-ACETAMINOPHN 37.5-32	284	0.03	1050691	96.86
HYDROCODONE/APAP 5/325 TAB	283	0.03	1050974	96.88
HYDROCODON-ACETAMINOPH 7.5-65	278	0.03	1051252	96.91
ROXICET 5 MG-325MG TABLET	277	0.03	1051529	96.94
OXYCODONE HCL 10 MG TABLET	270	0.02	1051799	96.96
VAZOTUSS HC SUSPENSION	269	0.02	1052068	96.99
ENTEX HC LIQUID	268	0.02	1052336	97.01
OXYCODONE-APAP 10-650 MG TAB	268	0.02	1052604	97.04
H-C TUSSIVE 5-2.5-2 SYRUP	261	0.02	1052865	97.06
METHADONE HCL 5 MG/5 ML SOLUT	259	0.02	1053124	97.08
LORTAB ELIXIR	256	0.02	1053380	97.11
ATUSS HD CAPSULE	255	0.02	1053635	97.13
GANI-TUSS-DM NR 100-10MG/5 LI	254	0.02	1053889	97.15
PNEUMOTUSSIN TABLET	254	0.02	1054143	97.18
HYDRO-PC II PLUS LIQUID	252	0.02	1054395	97.20
DURATUSS HD ELIXIR	249	0.02	1054644	97.22
HYDROCODONE BT-IBUPROFEN TAB	248	0.02	1054892	97.25
MEDTUSS HD ELIXIR	248	0.02	1055140	97.27
OXYCODON-ACETAMINOPHEN 7.5-32	248	0.02	1055388	97.29
HYDROCODON-ACETAMINOPHN 10-65	243	0.02	1055631	97.31
HALOTUSSIN-DAC SYRUP S/F	241	0.02	1055872	97.34
ENDOCET 10-650 MG TABLET	239	0.02	1056111	97.36
PANLOR SS TABLET	238	0.02	1056349	97.38
DEMEROL 50MG/5ML SYRUP	237	0.02	1056586	97.40
OXYCODONE-APAP 5-325 TABLET	237	0.02	1056823	97.42
ACETAMINOPHEN-COD #2 TABLET	231	0.02	1057054	97.45
XODOL 10/300 TABLET	230	0.02	1057284	97.47
HYDRON KGS LIQUID	229	0.02	1057513	97.49
BUTALBITAL COMPOUND TABLET	228	0.02	1057741	97.51
GUAIFENESIN-PE-HCOD SYRUP	228	0.02	1057969	97.53
PANLOR DC CAPSULE	228	0.02	1058197	97.55
MEPERIDINE 50 MG/5 ML SYRUP	225	0.02	1058422	97.57

HYDROCODON-ACETAMINOPH 2.5-50	224	0.02	1058646	97.59
DE-CHLOR G LIQUID	220	0.02	1058866	97.61
ACETAMINOPHEN/COD #4 TABLET	218	0.02	1059084	97.63
ROXICET 5/325 ORAL SOLUTION	216	0.02	1059300	97.65
DYTAN-HC SUSPENSION	215	0.02	1059515	97.67
OXYCODONE HCL 5MG TABLET	213	0.02	1059728	97.69
TRAMADOL-APAP 37.5-325 MG TAB	211	0.02	1059939	97.71
ACETAMINOPHEN-CODEINE 300MG-6	209	0.02	1060148	97.73
HC 2.5/PE 5 /DBROM 1 MG SYRUP	207	0.02	1060355	97.75
DE-CHLOR MR LIQUID	204	0.02	1060559	97.77
ENDOCET 10/650MG TABLET	199	0.02	1060758	97.79
HYDROCODONE-APAP 10-325 TABLE	196	0.02	1060954	97.80
PROPOXY-N/APAP 50-325 TAB	195	0.02	1061149	97.82
TUSSI-ORGANIDIN NR LIQUID	193	0.02	1061342	97.84
OXYCODONE 5 MG TABLET	192	0.02	1061534	97.86
ZAMICET SOLUTION	191	0.02	1061725	97.88
PROTUSS LIQUID	190	0.02	1061915	97.89
XODOL TABLET	190	0.02	1062105	97.91
PROMETHAZINE W/CODEINE SYRUP	189	0.02	1062294	97.93
COLDCOUGH HC LIQUID	187	0.02	1062481	97.95
ENDAL-HD LIQUID	186	0.02	1062667	97.96
OXYCODONE/APAP 5/500 CAP	186	0.02	1062853	97.98
HYDRON CP 10-5-2MG/5 SYRUP	185	0.02	1063038	98.00
DE-CHLOR HD LIQUID	184	0.02	1063222	98.01
DYTAN-HC SUSPENION	182	0.02	1063404	98.03
HYDRO-DP 7.5-2-12.5 SYRUP	182	0.02	1063586	98.05
MORPHINE SULFATE 15 MG TABLET	181	0.02	1063767	98.06
ZYMINE HC LIQUID	180	0.02	1063947	98.08
HYDRO GP LIQUID	179	0.02	1064126	98.10
OXYCODONE HCL 5 MG CAPSULE	177	0.02	1064303	98.11
LORTUSS HC LIQUID	176	0.02	1064479	98.13
BUTALBITAL COMPOUND CAPSULE	170	0.02	1064649	98.15
POLY HIST HC SOLUTION	169	0.02	1064818	98.16
OXYCODONE/APAP 7.5/500 TAB	168	0.02	1064986	98.18
ENDACOF-HC 30-1.7-2/5 SOLUTIO	167	0.02	1065153	98.19
DURADAL HD PLUS LIQUID	166	0.02	1065319	98.21
HYDROMORPHONE HCL 2 MG TABLET	166	0.02	1065485	98.22
PSE BPM HD SYRUP	166	0.02	1065651	98.24
HALOTUSSIN-DAC LIQUID S/F	163	0.02	1065814	98.25
DARVOCET-N 50 TABLET	162	0.01	1065976	98.27
DECOHISTINE DH LIQUID	161	0.01	1066137	98.28
DIHISTINE DH LIQUID	154	0.01	1066291	98.30
HYDROMORPHONE 2 MG TABLET	154	0.01	1066445	98.31
LORTAB 10-300/15 SOLUTION	153	0.01	1066598	98.33
POLY CS SYRUP	153	0.01	1066751	98.34
HYDROCODONE/GUAIFENESIN SYRUP	152	0.01	1066903	98.35
MORPHINE SULF 10 MG/5 ML SOLN	151	0.01	1067054	98.37
VI-Q-TUSS 100-5 MG/5 SYRUP	148	0.01	1067202	98.38
TUSDEC-HC LIQUID	147	0.01	1067349	98.39
PROPOXYPHENE/APAP 65/650 TB	146	0.01	1067495	98.41
ABER-TUSS HC SYRUP	142	0.01	1067637	98.42

GUIATUSS DAC 30-10-100 SYRUP	142	0.01	1067779	98.43
PHENYLHISTINE DH LIQUID	142	0.01	1067921	98.45
HYDROCODONE-ACETAMIN 10-325 M	141	0.01	1068062	98.46
GUIATUSS AC 100-10MG/5 SYRUP	140	0.01	1068202	98.47
MINTUSS HD LIQUID	139	0.01	1068341	98.49
PRO-COF SOLUTION	138	0.01	1068479	98.50
ROXICODONE 5MG/5ML SOLUTION	138	0.01	1068617	98.51
BUTALBITAL COMP/COD #3 CAP	137	0.01	1068754	98.52
VISVEX HC LIQUID	135	0.01	1068889	98.54
ACETAMINOPHEN/COD SOLUTION	133	0.01	1069022	98.55
OXYCODONE-ACETAMINOPHEN 10-65	132	0.01	1069154	98.56
PROMETHAZINE VC-CODEINE SYRUP	132	0.01	1069286	98.57
ROXICET 5-325 TABLET	131	0.01	1069417	98.58
CODICLEAR DH SYRUP	129	0.01	1069546	98.60
PHENYLEPH-HCOD BT-CP 10-5-2MG	129	0.01	1069675	98.61
ULTRACET 37.5-325MG TABLET	125	0.01	1069800	98.62
ACETAMINOPHEN/CODEINE SOLN	124	0.01	1069924	98.63
PROPOXYPHENE HCL 65MG CAP	122	0.01	1070046	98.64
ENDOCET 10MG-650MG TABLET	121	0.01	1070167	98.65
MORPHINE SULF 10MG/5ML SOLN	118	0.01	1070285	98.66
MEPERITAB 50 MG TABLET	115	0.01	1070400	98.68
RINDAL HD PLUS SYRUP	115	0.01	1070515	98.69
CYTUSS HC 5-2.5-2 SYRUP	113	0.01	1070628	98.70
OXYCODONE-APAP 7.5-325 MG TAB	112	0.01	1070740	98.71
BUTALB-CAFF-ACETAMINOPH-CODEI	111	0.01	1070851	98.72
ENDAL-HD PLUS SYRUP	111	0.01	1070962	98.73
HYDRO PC II 7.5-2-2/5 SYRUP	110	0.01	1071072	98.74
PERCOCET 10/650MG TABLET	110	0.01	1071182	98.75
TUSSINATE SYRUP	106	0.01	1071288	98.76
COLDCOUGH HC SYRUP	105	0.01	1071393	98.77
OXYCODONE-APAP 7.5-325 MG TB	105	0.01	1071498	98.78
CODIMAL DH SYRUP	103	0.01	1071601	98.79
HYDRO-TUSSIN DHC 15-7.5-2/5 S	103	0.01	1071704	98.80
DYNEX HD LIQUID	102	0.01	1071806	98.81
PROPOXYPH-ACETAMINOPHN 100-65	102	0.01	1071908	98.81
Q-V TUSSIN 30-2.5-2/5 SYRUP	101	0.01	1072009	98.82
MYPHETANE DC COUGH SYRUP	98	0.01	1072107	98.83
PERCOCET 10/325MG TABLET	98	0.01	1072205	98.84
TUSSIVE HC SYRUP	98	0.01	1072303	98.85
D-TANN HC SUSPENSION	97	0.01	1072400	98.86
TUSSIGON TABLET	96	0.01	1072496	98.87
OXYCODONE HCL 15 MG TABLET	95	0.01	1072591	98.88
HYDROCODONE-GUAIFENESIN SYRUP	94	0.01	1072685	98.89
MORPHINE SULFATE IR 15 MG TAB	94	0.01	1072779	98.89
ATUSS EX LIQUID	92	0.01	1072871	98.90
POT GUAIACO/HCOD BT LIQUID	87	0.01	1072958	98.91
GUAIFENESIN W/CODEINE SYRUP	85	0.01	1073043	98.92
OXYCODON HCL-APAP 10/325 MG T	84	0.01	1073127	98.93
DEMEROL 50MG TABLET	83	0.01	1073210	98.93
SU-TUSS HD 100-30-2.5 ELIXIR	83	0.01	1073293	98.94
GUAIF/P-EPHED/HYDROCO LIQ	82	0.01	1073375	98.95

NORCO 10/325 TABLET	82	0.01	1073457	98.96
BROMPHENEX HD SYRUP	81	0.01	1073538	98.96
COTUSS-V SYRUP	80	0.01	1073618	98.97
GUAIFENESIN-CODEINE SYRUP	80	0.01	1073698	98.98
VANEX HD SYRUP	80	0.01	1073778	98.99
EXETUSS-HC LIQUID	79	0.01	1073857	98.99
MINTUSS G 100-10-2/5 SYRUP	79	0.01	1073936	99.00
CODAL-DH SYRUP	78	0.01	1074014	99.01
HYDROCODONE BIT-IBUPROFEN TAB	78	0.01	1074092	99.02
ULTRAM ER 100 MG TABLET	78	0.01	1074170	99.02
ATUSS HX CAPSULE	77	0.01	1074247	99.03
BUTALBITAL/CAFF/APAP/COD CP	77	0.01	1074324	99.04
TYLENOL W/CODEINE #3 TABLET	77	0.01	1074401	99.04
BROMPHENIRAMINE-HYDROCOD-PSE	76	0.01	1074477	99.05
HYDRO-TUSSIN HD 100-30-2.5 SO	76	0.01	1074553	99.06
MORPHINE SULFATE 10 MG/5 ML S	76	0.01	1074629	99.07
GUAIFENESIN-CODEINE 100-10MG/	75	0.01	1074704	99.07
GUAIFENESIN DAC 30-10-100 SYR	73	0.01	1074777	99.08
CODITUSS DH SYRUP	72	0.01	1074849	99.09
HYCET 7.5-325/15 SOLUTION	72	0.01	1074921	99.09
MORPHINE SULFATE 15 MG TAB	71	0.01	1074992	99.10
ULTRAM ER 200 MG TABLET	71	0.01	1075063	99.11
HC 3.5 MG/GUAI 300 MG SYRUP	70	0.01	1075133	99.11
POLY-TUSSIN DHC LIQUID	69	0.01	1075202	99.12
OXYCODONE-APAP 10-325MG TAB	68	0.01	1075270	99.12
ROXICET 5-325 ORAL SOLUTION	68	0.01	1075338	99.13
HYDRO-TUSSIN HC 15-3-2/5 LIQU	67	0.01	1075405	99.14
NEO HC SYRUP	67	0.01	1075472	99.14
ENDODAN 4.88/325 TABLET	65	0.01	1075537	99.15
MAXIDONE 10/750MG TABLET	65	0.01	1075602	99.16
PANCOF HC SYRUP	65	0.01	1075667	99.16
OXYCODONE 5MG TABLET	64	0.01	1075731	99.17
H-C TUSSIVE-NR SYRUP	63	0.01	1075794	99.17
PROMETHAZINE VC W/COD SYRUP	63	0.01	1075857	99.18
CAPITAL WITH CODEINE SUSP	62	0.01	1075919	99.18
NARIZ HC LIQUID	61	0.01	1075980	99.19
XODOL 10/300 10MG-300MG TABLE	61	0.01	1076041	99.20
COLDCOUGH HCM SYRUP	60	0.01	1076101	99.20
HYDROCODONE/APAP 10/750 TABLE	60	0.01	1076161	99.21
MINTUSS HC 10-2.5-2/5 SYRUP	60	0.01	1076221	99.21
MAX HC LIQUID	59	0.01	1076280	99.22
MORPHINE SULFATE ER 15 MG TAB	59	0.01	1076339	99.22
ACETAMINOPHEN-COD #4 TABLET	58	0.01	1076397	99.23
OXYCODONE 5 MG CAPSULE	58	0.01	1076455	99.23
OXYCODONE HCL 5MG/5ML SOL	58	0.01	1076513	99.24
HYDROCODONE-APAP 7.5-325 TAB	56	0.01	1076569	99.24
MAXI-TUSS HC LIQUID	56	0.01	1076625	99.25
HYDROCOD/CARBINOX/PSEUD LIQ	55	0.01	1076680	99.25
QUINDAL HD LIQUID	55	0.01	1076735	99.26
ACTAGEN-C SYRUP	54	0.00	1076789	99.26
HYDRO-TUSSIN XP LIQUID	54	0.00	1076843	99.27
OXYCODONE HCL-APAP 10/650 TAB	54	0.00	1076897	99.27
PERCOCET 7.5/325MG TABLET	54	0.00	1076951	99.28
PNEUMOTUSSIN HC EXPECTORANT	54	0.00	1077005	99.28

BUTALB-ACETAMINOPH-CAFF-CODEI	53	0.00	1077058	99.29
OXYCODONE-ACETAMINOPHEN 7.5-5	53	0.00	1077111	99.29
PROPOXYPHENE NAPSYLATE-APAP 1	53	0.00	1077164	99.30
OXYCODONE 5MG CAPSULE	52	0.00	1077216	99.30
BUPRENORPHINE-NALOXONE 8 MG-2	51	0.00	1077267	99.31
DURAGANIDIN NR LIQUID	51	0.00	1077318	99.31
ENDOCET 7.5/500MG TABLET	51	0.00	1077369	99.32
TYLENOL W/CODEINE ELIXIR	51	0.00	1077420	99.32
ENDACOF-XP 200-2.5/5 SYRUP	50	0.00	1077470	99.33
FIORICET W/CODEINE CAPSULE	50	0.00	1077520	99.33
LORTAB 2.5/500 TABLET	50	0.00	1077570	99.34
NORCO 5/325 TABLET	50	0.00	1077620	99.34
NORCO 7.5/325 TABLET	50	0.00	1077670	99.35
PROPOXYPHENE HCL 65 MG CAP	50	0.00	1077720	99.35
SYNALGOS-DC CAPSULE	50	0.00	1077770	99.35
TYLOX 5/500 CAPSULE	50	0.00	1077820	99.36
BROMCOMP HC 30-2.5-3/5 SYRUP	49	0.00	1077869	99.36
HYDROCODONE BT-HOMATROPINE MB	49	0.00	1077918	99.37
HYDROMORPHONE HCL 4 MG TABLET	49	0.00	1077967	99.37
CODEINE SULFATE 30 MG TABLET	48	0.00	1078015	99.38
KWELCOF LIQUID	47	0.00	1078062	99.38
PHENYLEPHRINE HD LIQUID	47	0.00	1078109	99.39
COMBUNOX TABLET	46	0.00	1078155	99.39
ENDOCET 7.5-325 MG TABLET	46	0.00	1078201	99.39
GANI-TUSS NR 100-10MG/5 LIQUI	46	0.00	1078247	99.40
FLUTUSS HC LIQUID	45	0.00	1078292	99.40
HYDROCODONE-APAP 10-750 TABLE	45	0.00	1078337	99.41
NALEX EXPECTORANT	45	0.00	1078382	99.41
PROLEX DH SOLUTION	45	0.00	1078427	99.42
QUENDAL HD PLUS LIQUID	45	0.00	1078472	99.42
GUAIFENESIN/CODEINE TABLET	44	0.00	1078516	99.42
HYDRON KGS 300-5MG/5 SYRUP	44	0.00	1078560	99.43
MARCOF EXPECTORANT	44	0.00	1078604	99.43
OXYCODONE HCL-APAP 7.5/500 TA	44	0.00	1078648	99.44
PERCOCET 7.5/500MG TABLET	44	0.00	1078692	99.44
XPECT-HC TABLET	44	0.00	1078736	99.44
HY-KXP LIQUID	43	0.00	1078779	99.45
HYDROMORPHONE 4 MG TABLET	43	0.00	1078822	99.45
PHENYLHISTINE 30-10-100 SYRUP	43	0.00	1078865	99.46
VICOPROFEN 7.5-200 MG TABLET	43	0.00	1078908	99.46
DEMEROL 100MG TABLET	42	0.00	1078950	99.46
ED TUSS HC SYRUP	42	0.00	1078992	99.47
FLUTUSS XP LIQUID	42	0.00	1079034	99.47
ACETAMINOPHEN W/CODEINE 12-12	40	0.00	1079074	99.48
PENTAZOCINE/NALOXONE TABLET	40	0.00	1079114	99.48
ULTRAM 50 MG TABLET	40	0.00	1079154	99.48
ACETAMINOPHN-TRAMADOL 325-37.	39	0.00	1079193	99.49
ASCOMP W/CODEINE CAPSULE	39	0.00	1079232	99.49

OXYCODONE-APAP 7.5/325 MG TAB	39	0.00	1079271	99.49
ROBITUSSIN-DAC SYRUP	39	0.00	1079310	99.50
SIMUC-HD ELIXIR	39	0.00	1079349	99.50
DARVOCET-N 100 TABLET	38	0.00	1079387	99.50
DE-CHLOR NX LIQUID	37	0.00	1079424	99.51
HYDROCOD-HOMATROP 5-1.5 MG/5	37	0.00	1079461	99.51
METHADONE HCL 5 MG TABLET	37	0.00	1079498	99.51
PERCOCET 2.5/325MG TABLET	37	0.00	1079535	99.52
ROXICET 5-325/5 ML SOLUTION	37	0.00	1079572	99.52
VICODIN 5/500 TABLET	37	0.00	1079609	99.52
DRITUSS HD 100-30-2.5 ELIXIR	36	0.00	1079645	99.53
HYDROCODONE HD SYRUP	36	0.00	1079681	99.53
HYDRON EX 120-2.5/5 SYRUP	36	0.00	1079717	99.53
ATUSS G 100-10-2/5 SYRUP	35	0.00	1079752	99.54
ENDOCET 10-325 MG TABLET	35	0.00	1079787	99.54
HYDROCODONE CP SYRUP	35	0.00	1079822	99.54
PROPOXYPHENE-APAP 50-325 MG T	35	0.00	1079857	99.55
HISTINEX D LIQUID	34	0.00	1079891	99.55
HISTUSSIN D SOLUTION	34	0.00	1079925	99.55
HYDROMORPHONE 2MG TABLET	34	0.00	1079959	99.56
MEPERIDINE 100MG TABLET	34	0.00	1079993	99.56
METHADONE HCL 10 MG TABLET	34	0.00	1080027	99.56
MORPHINE SULFATE 30 MG TABLET	34	0.00	1080061	99.57
MARCOF SOLUTION	33	0.00	1080094	99.57
MORPHINE SULF ER 15 MG TABLET	33	0.00	1080127	99.57
MORPHINE SULFATE 15MG TAB	33	0.00	1080160	99.58
ATUSS HC 10-2.5-2/5 SYRUP	32	0.00	1080192	99.58
DONATUSSIN DC 50-7.5-2.5 SYRU	32	0.00	1080224	99.58
ENDOCET 7.5/325 MG TABLET	31	0.00	1080255	99.58
MS CONTIN 15MG TABLET SA	31	0.00	1080286	99.59
OXYCODONE-APAP 7.5-500 MG TAB	31	0.00	1080317	99.59
VISVEX HC 30-2.5-3/5 SYRUP	31	0.00	1080348	99.59
ATUSS HD 30-5-2MG CAP MPHASE	30	0.00	1080378	99.60
HYDROCODONE-APAP 5-325 TAB	30	0.00	1080408	99.60
ROBITUSSIN A-C SYRUP	30	0.00	1080438	99.60
TUSSI-ORGANIDIN-S NR LIQ	30	0.00	1080468	99.60
CRANTEX HC 100-7.5-5 SYRUP	29	0.00	1080497	99.61
ENDOCET 10/325 MG TABLET	29	0.00	1080526	99.61
ENDOCET 10MG-325MG TABLET	29	0.00	1080555	99.61
OXYCODONE HCL 30 MG TABLET	29	0.00	1080584	99.61
PHENERGAN W/CODEINE SYRUP	29	0.00	1080613	99.62
QUINDAL-HD PLUS LIQUID	29	0.00	1080642	99.62
TRIPROLIDINE-C SYRUP	29	0.00	1080671	99.62
ZERLOR TABLET	29	0.00	1080700	99.63
OXYCODONE HCL 10 MG ER TABLET	28	0.00	1080728	99.63
OXYCODONE/APAP 5/325 TABLET	28	0.00	1080756	99.63
CODEINE SULFATE 30MG TABLET	27	0.00	1080783	99.63
GUAIFENESIN/P-EPHED HCL/HCOD	27	0.00	1080810	99.64
GUIAPLEX HC LIQUID	27	0.00	1080837	99.64
HC TUSSIVE-D SOLUTION	27	0.00	1080864	99.64
HYDROCODONE/APAP 5/500 CAP	27	0.00	1080891	99.64
OXYCODONE-ACETAMINOPHEN 2.5-3	27	0.00	1080918	99.65
PHENYLEPH-HCOD BT-CP 5-5-2MG/	27	0.00	1080945	99.65
PROTUSS 300-5MG/5 SYRUP	27	0.00	1080972	99.65

TRI-VENT HC 30-5-2MG/5 LIQUID	27	0.00	1080999	99.65
HYDROCODONE-APAP 10/650 TAB	26	0.00	1081025	99.66
MSIR 15MG TABLET	26	0.00	1081051	99.66
NUCOTUSS PEDIATRIC EXPECT	26	0.00	1081077	99.66
POLY-HISTINE CS SYRUP	26	0.00	1081103	99.66
PRO-COF D LIQUID	26	0.00	1081129	99.66
ROXANOL 20MG/ML SOLUTION	26	0.00	1081155	99.67
SYMTAN A SUSPENSION	26	0.00	1081181	99.67
TALACEN CAPLET	26	0.00	1081207	99.67
TRIANT-HC LIQUID	26	0.00	1081233	99.67
TUSSADUR-HD LIQUID	26	0.00	1081259	99.68
ANEXSIA 7.5/325MG TABLET	25	0.00	1081284	99.68
MEPERITAB 50MG TABLET	25	0.00	1081309	99.68
TUSSIGON 5 MG-1.5MG TABLET	25	0.00	1081334	99.68
CARISOPRODOL CPD/CODEINE TB	24	0.00	1081358	99.69
ENDOCET 10/325MG TABLET	24	0.00	1081382	99.69
ENDOCET 7.5/500 MG TABLET	24	0.00	1081406	99.69
METHADONE 10 MG/5 ML SOLUTION	24	0.00	1081430	99.69
MORPHINE SULF 100 MG/5 ML SOL	24	0.00	1081454	99.69
PANLOR DC 16-356-30 CAPSULE	24	0.00	1081478	99.70
GUIATUSS AC 100-10MG/5 LIQUID	23	0.00	1081501	99.70
OXYCONTIN 20MG TABLET SA	23	0.00	1081524	99.70
VICODIN TUSS SYRUP	23	0.00	1081547	99.70
DILAUDID 2MG TABLET	22	0.00	1081569	99.71
M-OXY 5MG TABLET	22	0.00	1081591	99.71
MINTUSS HD 10-2.5-4 SYRUP	22	0.00	1081613	99.71
MORPHINE SULFATE 100 MG/5ML S	22	0.00	1081635	99.71
RINDAL HD PLUS 7.5-3.5-2 SYRU	22	0.00	1081657	99.71
BUTALBITAL COMPOUND-CODEINE 3	21	0.00	1081678	99.72
CODEINE SULFATE 15 MG TABLET	21	0.00	1081699	99.72
DARVOCET A500 TABLET	21	0.00	1081720	99.72
HYCODAN TABLET	21	0.00	1081741	99.72
MORPHINE SULF 20MG/5ML SOLN	21	0.00	1081762	99.72
MORPHINE SULFATE 30 MG TAB	21	0.00	1081783	99.72
PSE BPM HD 30-1.7-2/5 SOLUTIO	21	0.00	1081804	99.73
ASCOMP WITH CODEINE 30-50-325	20	0.00	1081824	99.73
DE-CHLOR MR 5-5-5MG/5 SYRUP	20	0.00	1081844	99.73
DONATUSSIN MAX LIQUID	20	0.00	1081864	99.73
HYDRO-PC II PLUS 7.5-3.5-2 SY	20	0.00	1081884	99.73
MAXI-TUSS HCX LIQUID	20	0.00	1081904	99.74
MINTUSS MS 10-5-2MG/5 SYRUP	20	0.00	1081924	99.74
MORPHINE SULFATE IR 30 MG TAB	20	0.00	1081944	99.74
MS CONTIN 30MG TABLET SA	20	0.00	1081964	99.74
OXYCODON-ACETAMINOPHEN 7.5-50	20	0.00	1081984	99.74
OXYCODONE/ASA 4.88/325 TAB	20	0.00	1082004	99.75
OXYCONTIN 10MG TABLET SA	20	0.00	1082024	99.75
PERCOCET 2.5/325 MG TABLET	20	0.00	1082044	99.75
QUINTEX HC LIQUID	20	0.00	1082064	99.75
COUGHTUSS 5-5-2MG/5 LIQUID	19	0.00	1082083	99.75
HYDROCET 5/500 CAPSULE	19	0.00	1082102	99.75
PANCOF XP SYRUP	19	0.00	1082121	99.76
PENTAZOCINE/ACETAMIN TABLET	19	0.00	1082140	99.76
XODOL 10-300 10MG-300MG TABLE	19	0.00	1082159	99.76
CAPITAL W-CODEINE 120-12MG/5	18	0.00	1082177	99.76

PANLOR SS 32-713-60 TABLET	18	0.00	1082195	99.76
HYDROCODONE-APAP 10-660 TABLE	17	0.00	1082212	99.76
HYDROCODONE/APAP 10/660 TAB	17	0.00	1082229	99.77
GUAIFEN-C LIQUID	16	0.00	1082245	99.77
MORPHINE SULF 15 MG TAB SA	16	0.00	1082261	99.77
TRAMADOL HCL-APAP 37.5-325 TA	16	0.00	1082277	99.77
ALAHIST DHC LIQUID	15	0.00	1082292	99.77
HC 2.5-PE 5-DBROM 1 MG SYRUP	15	0.00	1082307	99.77
METHADOSE 10 MG TABLET	15	0.00	1082322	99.77
MORPHINE SULF 20 MG/5 ML SOLN	15	0.00	1082337	99.78
PHENERGAN VC W/CODEINE SYR	15	0.00	1082352	99.78
PRO-COF 300-5MG/5 SYRUP	15	0.00	1082367	99.78
PROPOXYPHENE HCL 65 MG CAPSUL	15	0.00	1082382	99.78
ZAMICET 10-325/15 SOLUTION	15	0.00	1082397	99.78
BUTORPHANOL TARTRATE 10 MG/ML	14	0.00	1082411	99.78
CANGES-HC LIQUID	14	0.00	1082425	99.78
CYTUSS-HC NR SYRUP	14	0.00	1082439	99.79
DROTUSS-CP LIQUID	14	0.00	1082453	99.79
HYDROCODONE-APAP 7.5/750 TB	14	0.00	1082467	99.79
P-EPHED/HYDROCOD BIT/CP LIQ	14	0.00	1082481	99.79
POLY-TUSSIN DHC 4-7.5-3/5 LIQ	14	0.00	1082495	99.79
ROXICODONE 5 MG/5 ML SOLUTION	14	0.00	1082509	99.79
ZYDONE 10/400MG TABLET	14	0.00	1082523	99.79
BUPRENORPHINE HCL 8 MG TAB SU	13	0.00	1082536	99.79
BUTALBITAL COMP-CODEINE #3 CA	13	0.00	1082549	99.80
DE-CHLOR G 100-10-2/5 SYRUP	13	0.00	1082562	99.80
ENDOCET 7.5-500 MG TABLET	13	0.00	1082575	99.80
HYDROCOD-PHENYLE-CP LIQUID	13	0.00	1082588	99.80
LEVALL 5.0 LIQUID	13	0.00	1082601	99.80
MAXITUSS HC SYRUP	13	0.00	1082614	99.80
PNEUMOTUSSIN 200-2.5/5 SYRUP	13	0.00	1082627	99.80
QUAL-TUSSIN DC SYRUP	13	0.00	1082640	99.80
QUINTEX HC 100-7.5-5 SYRUP	13	0.00	1082653	99.81
Z-COF HC LIQUID	13	0.00	1082666	99.81
DE-CHLOR HD 10-2.5-4 SYRUP	12	0.00	1082678	99.81
HISTINEX D SOLUTION	12	0.00	1082690	99.81
HYCODAN SYRUP	12	0.00	1082702	99.81
HYDRO GP 50-7.5-2.5 SYRUP	12	0.00	1082714	99.81
HYDROCODONE GF SYRUP	12	0.00	1082726	99.81
LEVALL 5.0 100-15-5/5 SYRUP	12	0.00	1082738	99.81
LISINOPRIL 30 MG TABLET	12	0.00	1082750	99.81
MINTUSS MR LIQUID	12	0.00	1082762	99.82
MS CONTIN 60MG TABLET SA	12	0.00	1082774	99.82
OXYCODONE-APAP 7.5-325MG TB	12	0.00	1082786	99.82
POLY-TUSSIN AC LIQUID	12	0.00	1082798	99.82
ROXICODONE 5 MG TABLET	12	0.00	1082810	99.82
ANEXSIA 5/325MG TABLET	11	0.00	1082821	99.82
BROVEX HC 30-2.5-3/5 SYRUP	11	0.00	1082832	99.82
DILAUDID-5 1 MG/ML LIQUID	11	0.00	1082843	99.82
GUAIFENESIN-CARBETAPENTANE 60	11	0.00	1082854	99.82
LORTAB 7.5/500 TABLET	11	0.00	1082865	99.82
MEPERIDINE 100 MG TABLET	11	0.00	1082876	99.83

METHADONE HCL 10 MG/5 ML SOLU	11	0.00	1082887	99.83
NUCYNTA 50 MG TABLET	11	0.00	1082898	99.83
OXYCODON-ACETAMINOPHEN 2.5-32	11	0.00	1082909	99.83
PAREGORIC 2 MG/5 ML LIQUID	11	0.00	1082920	99.83
PERCOCET 2.5-325 MG TABLET	11	0.00	1082931	99.83
PERCOCET 5/325MG TABLET	11	0.00	1082942	99.83
POLYTINE CS SYRUP	11	0.00	1082953	99.83
PROLEX DH 300-5MG/5 SOLUTION	11	0.00	1082964	99.83
SUBOXONE 8 MG-2 MG TAB SUBL	11	0.00	1082975	99.83
TUSDEC-HC 7.5-3.75/5 LIQUID	11	0.00	1082986	99.84
ULTRAM ER 300 MG TABLET	11	0.00	1082997	99.84
Z-COF HCX LIQUID	11	0.00	1083008	99.84
ATUSS HS 30-5-4MG/5 ORAL SUSP	10	0.00	1083018	99.84
BROMPHENEX HD 30-1.7-2/5 SOLU	10	0.00	1083028	99.84
BUTALBITAL-CAFF-APAP-COD CAP	10	0.00	1083038	99.84
EXECOF-XP SYRUP	10	0.00	1083048	99.84
GUAIFENESIN-PHENYLEPHRINE-HCO	10	0.00	1083058	99.84
HYDROCODONE/HOMATROP SYRUP	10	0.00	1083068	99.84
LORTAB 5/500 TABLET	10	0.00	1083078	99.84
MEPERIDINE 50 MG/ML SOLUTION	10	0.00	1083088	99.85
MORPHINE SULF 30 MG TAB SA	10	0.00	1083098	99.85
MORPHINE SULFATE 30MG TAB	10	0.00	1083108	99.85
MORPHINE SULFATE ER 30 MG TAB	10	0.00	1083118	99.85
OXYCONTIN 40MG TABLET SA	10	0.00	1083128	99.85
OXYIR 5MG CAPSULE	10	0.00	1083138	99.85
P-EPD HCL/HCOD BT/CARBINOX 30	10	0.00	1083148	99.85
POLY-TUSSIN XP LIQUID	10	0.00	1083158	99.85
TALWIN NX TABLET	10	0.00	1083168	99.85
VOPAC TABLET	10	0.00	1083178	99.85
XODOL 10-300 TABLET	10	0.00	1083188	99.85
ASCOMP WITH CODEINE CAPSULE	9	0.00	1083197	99.86
BRONTEX TABLET	9	0.00	1083206	99.86
CHERATUSSIN AC 100-10MG/5 SYR	9	0.00	1083215	99.86
HYDROCODONE-POT GUAIACOSULFON	9	0.00	1083224	99.86
HYDROCODONE/APAP 7.5/325 TB	9	0.00	1083233	99.86
MEPERIDINE HCL 100 MG TABLET	9	0.00	1083242	99.86
ROBAFEN AC 100-10MG/5 LIQUID	9	0.00	1083251	99.86
ROXICODONE 30MG TABLET	9	0.00	1083260	99.86
SUBOXONE 8 MG-2 MG TABLET	9	0.00	1083269	99.86
TRIHIST-CS COUGH SYRUP	9	0.00	1083278	99.86
TUSSIVE HC 5-2.5-2 SYRUP	9	0.00	1083287	99.86
VICODIN ES TABLET	9	0.00	1083296	99.86
VORTEX SYRUP	9	0.00	1083305	99.87
XPECT-HC 600MG-5MG TAB.SR 12H	9	0.00	1083314	99.87
ZERLOR 32-713-60 TABLET	9	0.00	1083323	99.87
ZYDONE 5/400MG TABLET	9	0.00	1083332	99.87
B-TUSS 5-5-2MG/5 LIQUID	8	0.00	1083340	99.87
ENDOCET 7.5/325MG TABLET	8	0.00	1083348	99.87
EXECLEAR SYRUP	8	0.00	1083356	99.87
HC TUSSIVE-D 60-5MG/5ML SOLUT	8	0.00	1083364	99.87
HYDROCODONE-GUAIFENESIN 100-3	8	0.00	1083372	99.87

HYDROCODONE/GUAIFENESIN LIQ	8	0.00	1083380	99.87
LORTAB 7.5-500/15 SOLUTION	8	0.00	1083388	99.87
M-END 30-2.5-2/5 SYRUP	8	0.00	1083396	99.87
METHADONE 10MG/5ML SOLUTION	8	0.00	1083404	99.87
MORPHINE SULF 20 MG/ML SOLN	8	0.00	1083412	99.88
MORPHINE SULFATE IR 15 MG TB	8	0.00	1083420	99.88
OXYCODONE HCL 20 MG ER TABLET	8	0.00	1083428	99.88
OXYCODONE HCL-ACETAMINOPHEN 1	8	0.00	1083436	99.88
QUINDAL HD PLUS LIQUID	8	0.00	1083444	99.88
ROBAFEN AC SYRUP	8	0.00	1083452	99.88
SIMUC-HD 225-10-2.5 SOLUTION	8	0.00	1083460	99.88
TRAMADOL HCL ER 100 MG TABLET	8	0.00	1083468	99.88
TRIANT-HC 5-1.67-2/5 LIQUID	8	0.00	1083476	99.88
TUSSICAPS 10MG-8MG CAP ER 12H	8	0.00	1083484	99.88
TUSSICLEAR DH SYRUP	8	0.00	1083492	99.88
BUPRENORPHINE 8 MG TABLET SL	7	0.00	1083499	99.88
DARVOCET-N 50 50MG-325MG TABL	7	0.00	1083506	99.88
DARVON-N 100 MG TABLET	7	0.00	1083513	99.88
DIHISTINE DH 30-10-2/5 LIQUID	7	0.00	1083520	99.89
ED-TLC LIQUID	7	0.00	1083527	99.89
EXTENDRYL HC TABLET	7	0.00	1083534	99.89
GUAIFENESIN WITH CODEINE SYRU	7	0.00	1083541	99.89
HYDROCODONE BIT-HOMATROP SYRU	7	0.00	1083548	99.89
HYDROMORPHONE 4MG TABLET	7	0.00	1083555	99.89
IOTUSSIN HC SYRUP	7	0.00	1083562	99.89
LORTAB 10/500 TABLET	7	0.00	1083569	99.89
METHADONE HCL 10MG TABLET	7	0.00	1083576	99.89
METHADONE HCL 5MG TABLET	7	0.00	1083583	99.89
RHINACON DH LIQUID	7	0.00	1083590	99.89
ROXANOL 20 MG/ML SOLUTION	7	0.00	1083597	99.89
ROXICODONE 15MG TABLET	7	0.00	1083604	99.89
SPANTUSS HD ELIXIR	7	0.00	1083611	99.89
SUBOXONE 8 MG-2 MG TABLET SL	7	0.00	1083618	99.89
VICODIN HP TABLET	7	0.00	1083625	99.89
BALACET 325 TABLET	6	0.00	1083631	99.90
BUPRENORPHIN-NALOXON 8-2 MG S	6	0.00	1083637	99.90
BUTALBITAL-CAFF-APAP-COD CP	6	0.00	1083643	99.90
CAPITAL W/CODEINE 120-12MG/5	6	0.00	1083649	99.90
CARISOPRODOL COMPOUND-CODEINE	6	0.00	1083655	99.90
CODEINE SULFATE 15MG TABLET	6	0.00	1083661	99.90
DARVON-N 100MG TABLET	6	0.00	1083667	99.90
DILAUDID 4MG TABLET	6	0.00	1083673	99.90
DILAUDID-5 1MG/ML LIQUID	6	0.00	1083679	99.90
FIORINAL/CODEINE #3 CAPSULE	6	0.00	1083685	99.90
HYCET 7.5 MG-325 MG/15 ML SOL	6	0.00	1083691	99.90
HYCOMAL DH SYRUP	6	0.00	1083697	99.90
HYDROCODONE SYRUP	6	0.00	1083703	99.90
LORCET 5-325 MG TABLET	6	0.00	1083709	99.90
LORTUSS HC 7.5-3.75/5 LIQUID	6	0.00	1083715	99.90
METHADONE 5MG/5ML SOLUTION	6	0.00	1083721	99.90
MORPHINE SULF 30MG TAB SA	6	0.00	1083727	99.90

MORPHINE SULFATE 20 MG/5 ML S	6	0.00	1083733	99.90
NEO HC 7.5-5-3/5 LIQUID	6	0.00	1083739	99.91
OXYCODONE HCL 10 MG TAB.SR 12	6	0.00	1083745	99.91
OXYCODONE HCL 20 MG/ML ORAL C	6	0.00	1083751	99.91
OXYCODONE HCL 5MG CAPSULE	6	0.00	1083757	99.91
OXYCONTIN 40 MG TAB ER 12H	6	0.00	1083763	99.91
OXYCONTIN 80MG TABLET SA	6	0.00	1083769	99.91
PERCOCET 10MG-325MG TABLET	6	0.00	1083775	99.91
PERCOCET 10MG-650MG TABLET	6	0.00	1083781	99.91
PHENYLEPHRINE HD 5-2.5MG/5 LI	6	0.00	1083787	99.91
PNEUMOTUSSIN 300-2.5MG TABLET	6	0.00	1083793	99.91
PROMETHAZINE-CODEINE 10-6.25/	6	0.00	1083799	99.91
TUSSI-ORGANIDIN NR LIQ	6	0.00	1083805	99.91
TYLENOL-CODEINE NO.3 300MG-30	6	0.00	1083811	99.91
VIRTUSSIN DAC 30-10-100 SYRUP	6	0.00	1083817	99.91
ATUSS EX 120-2.5/5 SYRUP	5	0.00	1083822	99.91
B & O SUPPRETTES NO.15-A	5	0.00	1083827	99.91
BIOTUSSIN AC 100-10MG/5 LIQUI	5	0.00	1083832	99.91
BUTALBITAL COMPOUND 50-325-40	5	0.00	1083837	99.91
BUTALBITAL-CAF-APAP-COD CAP	5	0.00	1083842	99.91
C-COF XP LIQUID	5	0.00	1083847	99.92
COLDCOUGH HC 15-3-2/5 LIQUID	5	0.00	1083852	99.92
DARVON COMPOUND-65 PULVULE	5	0.00	1083857	99.92
ENDAL HD 7.5-2-12.5 SYRUP	5	0.00	1083862	99.92
GUAIFENESIN AC LIQUID	5	0.00	1083867	99.92
HC-PE-DBROM 5-2.5-1/5 LIQUID	5	0.00	1083872	99.92
HISTUSSIN HC 5-2.5-2 SYRUP	5	0.00	1083877	99.92
HYCOMINE COMPOUND TABLET	5	0.00	1083882	99.92
HYDROCODON-ACETAMINOPHN 10-66	5	0.00	1083887	99.92
HYDROMORPHONE HCL 8 MG TABLET	5	0.00	1083892	99.92
LORCET PLUS TABLET	5	0.00	1083897	99.92
MAXIDONE 10MG-750MG TABLET	5	0.00	1083902	99.92
MORPHINE SULF 15 MG TABLET CR	5	0.00	1083907	99.92
MORPHINE SULF 60 MG TAB SA	5	0.00	1083912	99.92
MORPHINE SULFATE IR 15MG TB	5	0.00	1083917	99.92
NALEX 200-60-5 EXPECT	5	0.00	1083922	99.92
NOVAHISTINE DH LIQUID	5	0.00	1083927	99.92
NUCOFED EXPECTORANT	5	0.00	1083932	99.92
NUCOTUSS EXPECTORANT	5	0.00	1083937	99.92
OPIUM TINCTURE	5	0.00	1083942	99.92
ORAMORPH SR 30MG TABLET SA	5	0.00	1083947	99.92
P-V-TUSSIN 60-5MG TABLET	5	0.00	1083952	99.92
PHENDACOF HC SYRUP	5	0.00	1083957	99.93
RINDAL HPD SYRUP	5	0.00	1083962	99.93
ULTRAM ER 200 MG TAB ER 24H	5	0.00	1083967	99.93
ABER-TUSS HC 10-2.5-2/5 SYRUP	4	0.00	1083971	99.93
BUPRENORPHINE-NALOXONE 2 MG-0	4	0.00	1083975	99.93
CODAFED PEDI EXPECTORANT	4	0.00	1083979	99.93
CODAMINE SYRUP	4	0.00	1083983	99.93
ENDOTUSS HD LIQUID	4	0.00	1083987	99.93
ENTEX HC 100-7.5-5 SYRUP	4	0.00	1083991	99.93
EXECLEAR 100-3.5/5 SYRUP	4	0.00	1083995	99.93
EXETUSS-HC 225-10-2.5 SOLUTIO	4	0.00	1083999	99.93

GUAIFENESIN-CODEINE 300MG-10M	4	0.00	1084003	99.93
HISTUSSIN HC 5-2.5-1/5 LIQUID	4	0.00	1084007	99.93
HYDRO-TUSSIN XP 100-15-3/5 LI	4	0.00	1084011	99.93
HYDROCODONE BIT-IBUPROFEN 7.5	4	0.00	1084015	99.93
HYDROCODONE BIT-POT GUAIACO L	4	0.00	1084019	99.93
HYDROCODONE PA SYRUP	4	0.00	1084023	99.93
HYDROMORPHONE HCL 1 MG/ML LIQ	4	0.00	1084027	99.93
LORTAB 10 MG-300 MG/15 ML ELX	4	0.00	1084031	99.93
METHADONE INTENSOL 10 MG/ML	4	0.00	1084035	99.93
METHADONE INTENSOL 10 MG/ML O	4	0.00	1084039	99.93
MINTUSS NX SYRUP	4	0.00	1084043	99.93
MORPHINE SULF 100 MG/5 ML CON	4	0.00	1084047	99.93
OXYCODONE HCL ER 80 MG TAB	4	0.00	1084051	99.93
PEDIATEX HC LIQUID	4	0.00	1084055	99.93
PENTAZOCINE-NALOXONE TABLET	4	0.00	1084059	99.93
PHENYLHISTINE DH 30-10-2/5 LI	4	0.00	1084063	99.94
POLY HIST HC 3.5-12.5/5 SOLUT	4	0.00	1084067	99.94
POLY-TUSSIN XP 200-10-5/5 LIQ	4	0.00	1084071	99.94
PROLEX DH 300-4.5/5 SYRUP	4	0.00	1084075	99.94
PROPOXY-N/APAP 100-500 TAB	4	0.00	1084079	99.94
REPREXAIN 5MG-200MG TABLET	4	0.00	1084083	99.94
SYNALGOS-DC 16-356-30 CAPSULE	4	0.00	1084087	99.94
TRAMADOL HCL ER 100 MG TAB ER	4	0.00	1084091	99.94
TUSSI-ORGANIDIN NR 100-10MG/5	4	0.00	1084095	99.94
TUSSICLEAR DH 100-3.5/5 SYRUP	4	0.00	1084099	99.94
TUSSINATE 5-3.5-12.5 SYRUP	4	0.00	1084103	99.94
TY-TAB W/CODEINE #3 TABLET	4	0.00	1084107	99.94
UNI TUSS HC 5-2.5-2 SYRUP	4	0.00	1084111	99.94
ALA-HIST AC LIQUID	3	0.00	1084114	99.94
BELLADONNA-OPIUM 30-16.2 MG S	3	0.00	1084117	99.94
BPM PE HC LIQUID	3	0.00	1084120	99.94
CARISOPRODOL CPD-CODEINE TAB	3	0.00	1084123	99.94
CODICLEAR DH 100-3.5/5 SYRUP	3	0.00	1084126	99.94
CODIMAL DH 5-1.66MG/5 SYRUP	3	0.00	1084129	99.94
CODITUSS DH 5-1.66MG/5 SYRUP	3	0.00	1084132	99.94
COLDCOUGH XP SYRUP	3	0.00	1084135	99.94
COTUSS-V 100-5MG/5 SYRUP	3	0.00	1084138	99.94
DE-CHLOR NX 150-3MG/5 SYRUP	3	0.00	1084141	99.94
DEMEROL 50 MG/5 ML SOLUTION	3	0.00	1084144	99.94
DEMEROL 50 MG/5 ML SYRUP	3	0.00	1084147	99.94
DEX-TUSS 300-10MG/5 LIQUID	3	0.00	1084150	99.94
DICOMAL-DH SYRUP	3	0.00	1084153	99.94
ENDACOF-TAB TABLET	3	0.00	1084156	99.94
ENDOCET 2.5-325 MG TABLET	3	0.00	1084159	99.94
ENDODAN 4.5-325MG TABLET	3	0.00	1084162	99.94
ETH-OXYDOSE 20 MG/ML SOLUTION	3	0.00	1084165	99.94
FIORICET W/CODEINE 30-50-325	3	0.00	1084168	99.94
GUIAPHEN HD SYRUP	3	0.00	1084171	99.95
HYDROCET 5 MG-500MG CAPSULE	3	0.00	1084174	99.95
HYDROCODONE-ACETAMINOPHEN 5MG	3	0.00	1084177	99.95
HYDROCODONE-IBUPROFEN 5MG-200	3	0.00	1084180	99.95

KWELCOF 100-5 MG/5 SYRUP	3	0.00	1084183	99.95
LORCET-HD CAPSULE	3	0.00	1084186	99.95
MARCOF 350-5MG/5 SYRUP	3	0.00	1084189	99.95
MAX HC 8-5-4MG/5 LIQUID	3	0.00	1084192	99.95
MAXI-TUSS HCX 12-6-2MG/5 LIQU	3	0.00	1084195	99.95
METHADOSE 5 MG TABLET	3	0.00	1084198	99.95
MINTUSS EX SYRUP	3	0.00	1084201	99.95
MORPHINE SULF 15MG TAB SA	3	0.00	1084204	99.95
MORPHINE SULF 30 MG TABLET CR	3	0.00	1084207	99.95
MS CONTIN 100MG TABLET SA	3	0.00	1084210	99.95
MS CONTIN 15 MG TABLET SA	3	0.00	1084213	99.95
MSIR 30MG TABLET	3	0.00	1084216	99.95
NORCO 10MG-325MG TABLET	3	0.00	1084219	99.95
NUCYNTA 75 MG TABLET	3	0.00	1084222	99.95
ORAMORPH SR 100MG TABLET SA	3	0.00	1084225	99.95
ORAMORPH SR 15MG TABLET SA	3	0.00	1084228	99.95
OXYCODONE HCL 10 MG TAB SA	3	0.00	1084231	99.95
OXYCODONE HCL 40 MG ER TABLET	3	0.00	1084234	99.95
OXYCODONE HCL ER 10 MG TAB ER	3	0.00	1084237	99.95
OXYCODONE-ACETAMINOPHEN 5MG-3	3	0.00	1084240	99.95
OXYCODONE-APAP 2.5-325 MG TAB	3	0.00	1084243	99.95
OXYCODONE-ASPIRIN 4.5-325MG T	3	0.00	1084246	99.95
OXYTOCIN 10 UNITS/ML VIAL	3	0.00	1084249	99.95
PENTAZOCINE-NALOXONE HCL 50MG	3	0.00	1084252	99.95
PERCOCET 5-325 MG TABLET	3	0.00	1084255	99.95
PERCOCET 7.5-325 MG TABLET	3	0.00	1084258	99.95
POLY-TUSSIN AC 4-7.5-10/5 LIQ	3	0.00	1084261	99.95
PROPOXYPHENE COMP-65 CAP	3	0.00	1084264	99.95
PROPOXYPHENE HCL-ACETAMINOPHE	3	0.00	1084267	99.95
PROPOXYPHENE NAPSYLATE W/APAP	3	0.00	1084270	99.95
PROPOXYPHENE-APAP 65-650 MG T	3	0.00	1084273	99.95
PSEUDATEX HC LIQUID	3	0.00	1084276	99.95
REPREXAIN 5-200 MG TABLET	3	0.00	1084279	99.96
ROXIDOCONE 5MG TABLET	3	0.00	1084282	99.96
ROXIPRIN 4.88/325 TABLET	3	0.00	1084285	99.96
RYZOLT ER 100 MG TABLET	3	0.00	1084288	99.96
SYNALGOS-DC CAPSULE	3	0.00	1084291	99.96
TUSANA-D 12-6-2MG/5 LIQUID	3	0.00	1084294	99.96
TUSSICAPS 10 MG/8 MG CAPSULE	3	0.00	1084297	99.96
TYLENOL WITH CODEINE #3 TABLE	3	0.00	1084300	99.96
VAZOTUSS HC 10-5-6MG/5 ORAL S	3	0.00	1084303	99.96
ZOLVIT 10-300/15 SOLUTION	3	0.00	1084306	99.96
ZUTRIPRO 5-4-60MG/5 SOLUTION	3	0.00	1084309	99.96
ZYDONE 10MG-400MG TABLET	3	0.00	1084312	99.96
ZYMINE HC 30-2.5MG/5 LIQUID	3	0.00	1084315	99.96
A-COF DH SYRUP	2	0.00	1084317	99.96
ACETAMINOPHEN W/CODEINE 300MG	2	0.00	1084319	99.96
ALA-HIST AC 7.5-10MG/5 LIQUID	2	0.00	1084321	99.96
ALAHIST DHC 7.5-3MG/5 LIQUID	2	0.00	1084323	99.96
ANEXSIA 7.5-325MG TABLET	2	0.00	1084325	99.96

ATUSS HX 300MG-5MG CAP MPHASE	2	0.00	1084327	99.96
BPM PE HC 7.5-2.5-4 SOLUTION	2	0.00	1084329	99.96
BROMPHEN-DC COUGH SYRUP	2	0.00	1084331	99.96
BUPRENORPHIN-NALOXON 8-2 MG T	2	0.00	1084333	99.96
BUTALBITAL-CAFF-APAP-CODEINE	2	0.00	1084335	99.96
CANGES-XP LIQUID	2	0.00	1084337	99.96
CO-GESIC 5 MG-500MG TABLET	2	0.00	1084339	99.96
CO-GESIC 5/500 TABLET	2	0.00	1084341	99.96
CODAFED 30-10-100 SYRUP	2	0.00	1084343	99.96
CODEINE SULFATE 60 MG TABLET	2	0.00	1084345	99.96
COLDCOUGH HCM 15-3MG/5ML SYRU	2	0.00	1084347	99.96
COMBUNOX 400MG-5MG TABLET	2	0.00	1084349	99.96
CORDRON-HC 17.5-1.67 LIQUID	2	0.00	1084351	99.96
COTABFLU 4-20-500MG TABLET	2	0.00	1084353	99.96
D-TANN HC 7.5-3.5-25 ORAL SUS	2	0.00	1084355	99.96
DARVOCET A500 100-500MG TABLE	2	0.00	1084357	99.96
DARVOCET-N 100 100-650MG TABL	2	0.00	1084359	99.96
DARVON 65MG PULVULE	2	0.00	1084361	99.96
DEMEROL 50 MG TABLET	2	0.00	1084363	99.96
DILAUDID 1 MG/ML LIQUID	2	0.00	1084365	99.96
DONATUSS XP 180-15-30 ORAL SU	2	0.00	1084367	99.96
DONATUSSIN DC 120-6-2.5 SYRUP	2	0.00	1084369	99.96
DURADAL HD 5-1.67-2/5 SYRUP	2	0.00	1084371	99.96
DYNEX HD 200-45-6/5 SYRUP	2	0.00	1084373	99.96
DYTAN-HC 7.5-3.5-25 ORAL SUSP	2	0.00	1084375	99.96
ED-TLC 5-1.67-2/5 SYRUP	2	0.00	1084377	99.96
ENDAL-HD PLUS 7.5-3.5-2 SYRUP	2	0.00	1084379	99.96
ETH-OXYDOSE 20 MG/ML ORAL CON	2	0.00	1084381	99.96
EXECOF-XP 90-3MG/5ML SYRUP	2	0.00	1084383	99.96
FLUTUSS HC 7.5-2.5-2 LIQUID	2	0.00	1084385	99.96
HY-KXP 300-4.5/5 SYRUP	2	0.00	1084387	99.96
HYCODAN 5 MG-1.5MG TABLET	2	0.00	1084389	99.97
HYDROCODON-ACETAMINOPH 2.5-32	2	0.00	1084391	99.97
HYDROCODONE COMPOUND 5-1.5 MG	2	0.00	1084393	99.97
HYDROCODONE PA PEDI SYRUP	2	0.00	1084395	99.97
HYDROCODONE W/ACETAMINOPHEN 7	2	0.00	1084397	99.97
HYDROCODONE-ACETAMIN 2.5-325	2	0.00	1084399	99.97
HYDROCODONE-APAP 5-500 CAP	2	0.00	1084401	99.97
HYDROCODONE-HOMATROPINE TAB	2	0.00	1084403	99.97
HYDROCODONE/PE/CPM LIQUID	2	0.00	1084405	99.97
HYDROMORPHONE 0.5 MG/0.5 ML	2	0.00	1084407	99.97
HYDROMORPHONE HCL 4MG TABLET	2	0.00	1084409	99.97
IOTUSSIN D LIQUID	2	0.00	1084411	99.97
LORTAB 2.5-500MG TABLET	2	0.00	1084413	99.97
LORTAB 5-500 TABLET	2	0.00	1084415	99.97
LORTAB 7.5-500 MG/15 ML ELIXI	2	0.00	1084417	99.97
M-END LIQUID	2	0.00	1084419	99.97
M-END MAX LIQUID	2	0.00	1084421	99.97

MAXIDONE 10/750 MG TABLET	2	0.00	1084423	99.97
MEPERITAB 100MG TABLET	2	0.00	1084425	99.97
METHADOSE 40 MG TABLET DISPR	2	0.00	1084427	99.97
MINTUSS MR 5-5-5MG/5 SYRUP	2	0.00	1084429	99.97
MINTUSS NX 150-3MG/5 SYRUP	2	0.00	1084431	99.97
MORPHINE SULF 100 MG TABLET C	2	0.00	1084433	99.97
MORPHINE SULF 20MG/ML SOLN	2	0.00	1084435	99.97
MORPHINE SULF ER 30 MG TABLET	2	0.00	1084437	99.97
MORPHINE SULFATE ER 60 MG TAB	2	0.00	1084439	99.97
MSIR 15MG CAPSULE	2	0.00	1084441	99.97
NALEX DH 5-1.67MG/5 SYRUP	2	0.00	1084443	99.97
NALEX DH 5-2.5MG/5 LIQUID	2	0.00	1084445	99.97
NORCO 7.5-325MG TABLET	2	0.00	1084447	99.97
NOVAGEST DH LIQUID	2	0.00	1084449	99.97
NOVAHISTINE 30-10-100 SYRUP	2	0.00	1084451	99.97
NOVAHISTINE DH 30-10-2/5 LIQU	2	0.00	1084453	99.97
ORAMORPH SR 60MG TABLET SA	2	0.00	1084455	99.97
OXYCODONE CONC 20 MG/ML SOLN	2	0.00	1084457	99.97
OXYCODONE HCL 100 MG/5 ML SOL	2	0.00	1084459	99.97
OXYCODONE HCL 20 MG TAB.SR 12	2	0.00	1084461	99.97
OXYCODONE HCL ER 20 MG TAB ER	2	0.00	1084463	99.97
OXYCODONE HCL-IBUPROFEN 400MG	2	0.00	1084465	99.97
OXYCONTIN 10 MG TAB ER 12H	2	0.00	1084467	99.97
OXYCONTIN 40 MG TABLET SA	2	0.00	1084469	99.97
OXYCONTIN 80 MG TAB ER 12H	2	0.00	1084471	99.97
OXYFAST 20MG/ML SOLUTION	2	0.00	1084473	99.97
OXYTOCIN 10 UNIT/ML VIAL	2	0.00	1084475	99.97
PANCOF HC 15-3MG/5ML SYRUP	2	0.00	1084477	99.97
PERCOCET TABLET	2	0.00	1084479	99.97
PERCODAN TABLET	2	0.00	1084481	99.97
PERLOXX 10/300 MG TABLET	2	0.00	1084483	99.97
POLY-TUSSIN HD SYRUP	2	0.00	1084485	99.97
POLY-TUSSIN XP 200-60-5 EXPEC	2	0.00	1084487	99.97
POLY-TUSSIN XP EXPECTORANT	2	0.00	1084489	99.97
PRO-COF D 300-30-5/5 LIQUID	2	0.00	1084491	99.97
PROMETHAZINE VC-CODEINE 5-10-	2	0.00	1084493	99.97
PROPOXYPH-ACETAMINOPHEN 50-32	2	0.00	1084495	99.97
QUAL-TUSSIN DC 50-7.5-2.5 SYR	2	0.00	1084497	99.98
RELACON-HC NR 8-3.5-2.5 SYRUP	2	0.00	1084499	99.98
ROXANOL 100MG/5ML SOLUTION	2	0.00	1084501	99.98
STAGESIC 5-500 CAPSULE	2	0.00	1084503	99.98
STAGESIC 5/500 CAPSULE	2	0.00	1084505	99.98
SUBOXONE 2 MG-0.5 MG TABLET S	2	0.00	1084507	99.98
TRAMADOL HCL ER 200 MG TAB ER	2	0.00	1084509	99.98
TRICODE GF 30-8-200/5 LIQUID	2	0.00	1084511	99.98
TUSANA-D LIQUID	2	0.00	1084513	99.98
TUSSICAPS 5MG-4MG CAP ER 12H	2	0.00	1084515	99.98
TUSSIDEN C 300-10MG/5 LIQUID	2	0.00	1084517	99.98
TUSSIDEN C LIQUID	2	0.00	1084519	99.98
TUSSIGON 5-1.5MG TABLET	2	0.00	1084521	99.98
TYLENOL W/CODEINE #2 TABLET	2	0.00	1084523	99.98
TYLENOL W/CODEINE NO.3 300MG-	2	0.00	1084525	99.98
TYLOX 5 MG-500MG CAPSULE	2	0.00	1084527	99.98
ULTRAM ER 100 MG TAB ER 24H	2	0.00	1084529	99.98

UNI TUSS HC SYRUP S/F	2	0.00	1084531	99.98
VICODIN 5-500 TABLET	2	0.00	1084533	99.98
ZYDONE 7.5/400MG TABLET	2	0.00	1084535	99.98
A-COF DH 300-3.5/5 LIQUID	1	0.00	1084536	99.98
ACETAMINOPHEN-CODEINE 300MG/1	1	0.00	1084537	99.98
ACETAMINOPHEN-CODEINE 360-36/	1	0.00	1084538	99.98
ACETAMINOPHEN-CODEINE SOLN	1	0.00	1084539	99.98
ACTIQ 200MCG LOZENGE	1	0.00	1084540	99.98
ACTIQ 400MCG LOZENGE	1	0.00	1084541	99.98
ANEXSIA 5/500 TABLET	1	0.00	1084542	99.98
APAP-CAFFEINE-DIHYDROCODE TAB	1	0.00	1084543	99.98
AVINZA 120 MG CAPSULE	1	0.00	1084544	99.98
AVINZA 30 MG CAPSULE	1	0.00	1084545	99.98
AVINZA 30 MG CPMP 24HR	1	0.00	1084546	99.98
AVINZA 90MG CAPSULE	1	0.00	1084547	99.98
B & O SUPPRETTES NO.15-A 30-1	1	0.00	1084548	99.98
B & O SUPPRETTES NO.16-A	1	0.00	1084549	99.98
B & O SUPPRETTES NO.16-A 60-1	1	0.00	1084550	99.98
BALACET 325 100-325MG TABLET	1	0.00	1084551	99.98
BROMODIPHENHYD/COD SYRUP	1	0.00	1084552	99.98
BROMPHENIRAMINE DC SYRUP	1	0.00	1084553	99.98
BRONTEX 150-5/10ML LIQUID	1	0.00	1084554	99.98
BRONTEX LIQUID	1	0.00	1084555	99.98
BUPRENORPHINE HCL 2 MG TAB SU	1	0.00	1084556	99.98
BUPRENORPHINE-NALOXONE 12 MG-	1	0.00	1084557	99.98
BUPRENORPHN-NALOXN 2-0.5 MG S	1	0.00	1084558	99.98
BUTALBITAL COMPOUND 325-40-50	1	0.00	1084559	99.98
C-COF HC LIQUID	1	0.00	1084560	99.98
CANGES-HC 7.5-2.5-2 LIQUID	1	0.00	1084561	99.98
CANGES-HC NR 7.5-2.5-4 SOLUTI	1	0.00	1084562	99.98
CODAL-DH 5-1.66MG/5 SYRUP	1	0.00	1084563	99.98
CODEINE 10 MG-GUAI 300 MG LIQ	1	0.00	1084564	99.98
CODEINE 10 MG/GUAI 300 MG LIQ	1	0.00	1084565	99.98
CODEINE PHOSPHATE 15MG/5ML SO	1	0.00	1084566	99.98
CODEINE SULFATE 30 MG/5 ML SO	1	0.00	1084567	99.98
CODICLEAR DH 100-5MG/5 SYRUP	1	0.00	1084568	99.98
COMBIFLEX ES 500-500-20 TABLE	1	0.00	1084569	99.98
COMBIFLEX ES TABLET	1	0.00	1084570	99.98
CYNDAL EXPECTORANT	1	0.00	1084571	99.98
DARVOCET-N 100 100-650 MG TAB	1	0.00	1084572	99.98
DARVON 65 MG PULVULE	1	0.00	1084573	99.98
DARVON 65MG CAPSULE	1	0.00	1084574	99.98
DEMEROL 100 MG TABLET	1	0.00	1084575	99.98
DETUSS 30-5-2MG/5 LIQUID	1	0.00	1084576	99.98
DIABETIC TUSSIN C 200-10MG/5	1	0.00	1084577	99.98
DIHISTINE EXPECTORANT	1	0.00	1084578	99.98
DILAUDID COUGH SYRUP	1	0.00	1084579	99.98
DONATUSSIN MAX 6-5-2MG/5 LIQU	1	0.00	1084580	99.98
DROCON-CS 30-2.5-2/5 SYRUP	1	0.00	1084581	99.98
DUOHIST DH 5-7.25-2/5 LIQUID	1	0.00	1084582	99.98
DURATUSS HD 225-10-2.5 SOLUTI	1	0.00	1084583	99.98
ED TUSS HC 10-2.5-4 SYRUP	1	0.00	1084584	99.98
EFASIN HD PLUS LIQUID	1	0.00	1084585	99.98

ENDOCET 5MG-325MG TABLET	1	0.00	1084586	99.98
ENDOCODONE 5MG TABLET	1	0.00	1084587	99.98
ENDODAN 4.8355-325 TABLET	1	0.00	1084588	99.98
ENTEX HC 22.5-3.75 LIQUID	1	0.00	1084589	99.98
ENTUSS EXPECTORANT	1	0.00	1084590	99.98
ENTUSS-D LIQUID	1	0.00	1084591	99.98
EXCOF LIQUID	1	0.00	1084592	99.98
EXCOF-SF LIQUID	1	0.00	1084593	99.98
EXECLEAR-C 200-10MG/5 LIQUID	1	0.00	1084594	99.98
FENTANYL CITRATE 600 MCG LOZE	1	0.00	1084595	99.98
FENTUSS EXPECTORANT	1	0.00	1084596	99.98
FIORICET-COD 50-300-40-30 CAP	1	0.00	1084597	99.98
FIORINAL W/CODEINE #3 30-50-3	1	0.00	1084598	99.98
FIORTAL/CODEINE #3 CAPSULE	1	0.00	1084599	99.98
FLUTUSS HC 7.5-2.5-4 SOLUTION	1	0.00	1084600	99.98
GESTUSS-HC SYRUP	1	0.00	1084601	99.98
GUAIFEN-C 150-5/10ML LIQUID	1	0.00	1084602	99.98
GUAIFENESIN W-CODEINE LIQ	1	0.00	1084603	99.98
GUAIFENESIN W/DEXTROMETHORPHA	1	0.00	1084604	99.98
GUAIFENESIN-CODEINE 300-10MG/	1	0.00	1084605	99.99
GUIATUSSIN DAC 30-10-100 SYRU	1	0.00	1084606	99.99
GUIATUSSIN W/CODEINE SYRUP	1	0.00	1084607	99.99
H-C TUSSIVE 5-2.5-1/5 LIQUID	1	0.00	1084608	99.99
HALOTUSSIN-DAC 100-30-10 SYRU	1	0.00	1084609	99.99
HALOTUSSIN-DAC LIQUID	1	0.00	1084610	99.99
HC 3.5 MG-GUAI 300 MG SYRUP	1	0.00	1084611	99.99
HC TUSSIVE-D SYRUP	1	0.00	1084612	99.99
HISTUSSIN D 60-5MG/5ML SOLUTI	1	0.00	1084613	99.99
HISTUSSIN D LIQUID	1	0.00	1084614	99.99
HYCODAN 5-1.5 MG/5 SYRUP	1	0.00	1084615	99.99
HYCOMINE COMPOUND TABLET	1	0.00	1084616	99.99
HYCOMINE PEDIATRIC SYRUP	1	0.00	1084617	99.99
HYDROCODON-ACETAMINOPHEN 5-30	1	0.00	1084618	99.99
HYDROCODONE W/ACETAMINOPHEN 5	1	0.00	1084619	99.99
HYDROCODONE-ACETAMINOPHEN 10-	1	0.00	1084620	99.99
HYDROCODONE-APAP 5/325 TABLET	1	0.00	1084621	99.99
HYDROCODONE-APAP 7.5-325 TB	1	0.00	1084622	99.99
HYDROMORPHONE 1 MG/ML SOLUTIO	1	0.00	1084623	99.99
HYDROMORPHONE 1MG/ML SOLN	1	0.00	1084624	99.99
HYDROMORPHONE 8 MG TABLET	1	0.00	1084625	99.99
HYDROMORPHONE HCL 2MG TABLET	1	0.00	1084626	99.99
HYDROMORPHONE HCL 8 MG TAB	1	0.00	1084627	99.99
HYDROMORPHONE HCL 8MG TAB	1	0.00	1084628	99.99
HYDROMORPHONE HCL ER 16 MG TA	1	0.00	1084629	99.99
HYDROPHENE DH SYRUP	1	0.00	1084630	99.99
HYDROTROPINE 5/1.5 SYRUP	1	0.00	1084631	99.99
IBUDONE 5-200 MG TABLET	1	0.00	1084632	99.99
IBUDONE 5MG-200MG TABLET	1	0.00	1084633	99.99
KADIAN 20 MG CAP SR PEL	1	0.00	1084634	99.99

KADIAN 30MG CAPSULE SR	1	0.00	1084635	99.99
KADIAN 60 MG CAP SR PEL	1	0.00	1084636	99.99
LORCET 10-650 10MG-650MG TABL	1	0.00	1084637	99.99
LORCET 10/650 TABLET	1	0.00	1084638	99.99
LORCET HD 5MG-500MG CAPSULE	1	0.00	1084639	99.99
LORCET PLUS 7.5-650 MG TABLET	1	0.00	1084640	99.99
LORTAB 10-500 TABLET	1	0.00	1084641	99.99
LORTAB 7.5-500 TABLET	1	0.00	1084642	99.99
M-END MAX 7.5-5-2/5 LIQUID	1	0.00	1084643	99.99
MAXI-TUSS HC 10-2.5-4 SYRUP	1	0.00	1084644	99.99
MAXIFED CDX 60-20-400 TABLET	1	0.00	1084645	99.99
MEPERIDINE-PROMETHAZINE 50 MG	1	0.00	1084646	99.99
MEPROZINE 50 MG-25MG CAPSULE	1	0.00	1084647	99.99
METHADONE 40 MG TABLET DISPR	1	0.00	1084648	99.99
METHADONE 40MG TABLET DISPR	1	0.00	1084649	99.99
METHADONE HCL 10 MG/ML ORAL C	1	0.00	1084650	99.99
METHADONE HCL 40 MG DISKET	1	0.00	1084651	99.99
METHADONE HCL 40MG TABLET SOL	1	0.00	1084652	99.99
METHADONE INTENSOL 10MG/ML	1	0.00	1084653	99.99
MINTEX HC 30-5-2MG/5 LIQUID	1	0.00	1084654	99.99
MINTUSS EX 120-2.5/5 SYRUP	1	0.00	1084655	99.99
MORPHINE SULF 60MG TAB SA	1	0.00	1084656	99.99
MORPHINE SULF CR 15 MG TABLET	1	0.00	1084657	99.99
MORPHINE SULF ER 100 MG TAB	1	0.00	1084658	99.99
MORPHINE SULF ER 60 MG TABLET	1	0.00	1084659	99.99
MORPHINE SULFATE 10 MG SUPP.R	1	0.00	1084660	99.99
MORPHINE SULFATE 10 MG TAB	1	0.00	1084661	99.99
MORPHINE SULFATE 100 MG TABLE	1	0.00	1084662	99.99
MORPHINE SULFATE 10MG TAB	1	0.00	1084663	99.99
MORPHINE SULFATE 20 MG/ML SOL	1	0.00	1084664	99.99
MORPHINE SULFATE 50 MG/50ML P	1	0.00	1084665	99.99
MORPHINE SULFATE 60 MG TABLET	1	0.00	1084666	99.99
MORPHINE SULFATE IR 30MG TB	1	0.00	1084667	99.99
MS CONTIN 30 MG TABLET SA	1	0.00	1084668	99.99
MS CONTIN 60 MG TABLET SA	1	0.00	1084669	99.99
MSIR 10MG/5ML SOLUTION	1	0.00	1084670	99.99
MSIR 15 MG TABLET	1	0.00	1084671	99.99
MSIR 20MG/5ML SOLUTION	1	0.00	1084672	99.99
NAZARIN HC 200-2.5/5 LIQUID	1	0.00	1084673	99.99
NINJACOF-XG 8-200 MG/5 LIQUID	1	0.00	1084674	99.99
NORCO 5 MG-325MG TABLET	1	0.00	1084675	99.99
NOTUSS PD 5-4-2MG/5 LIQUID	1	0.00	1084676	99.99
NUCOFED 60-20-200 SYRUP	1	0.00	1084677	99.99
OPIUM TINCTURE 10 MG/ML	1	0.00	1084678	99.99
ORAMORPH SR 15 MG TABLET SA	1	0.00	1084679	99.99
ORAMORPH SR 60 MG TABLET SA	1	0.00	1084680	99.99
OXAYDO 5 MG TABLET ORL	1	0.00	1084681	99.99
OXYCODONE HCL 10MG TAB.SR 12H	1	0.00	1084682	99.99
OXYCODONE HCL 20 MG TABLET	1	0.00	1084683	99.99
OXYCODONE HCL 20 MG/ML SOL	1	0.00	1084684	99.99
OXYCODONE HCL 20 MG/ML SOLN	1	0.00	1084685	99.99
OXYCODONE HCL 40 MG TAB ER 12	1	0.00	1084686	99.99
OXYCODONE HCL 40MG TAB.SR 12H	1	0.00	1084687	99.99
OXYCODONE HCL 80 MG TAB.SR 12	1	0.00	1084688	99.99

OXYCODONE HCL ER 10 MG TABLET	1	0.00	1084689	99.99
OXYCODONE HCL ER 30 MG TAB ER	1	0.00	1084690	99.99
OXYCODONE HCL ER 80MG TAB	1	0.00	1084691	99.99
OXYCODONE HYDROCHLORIDE 100 %	1	0.00	1084692	99.99
OXYCODONE-APAP 10-650 TABLET	1	0.00	1084693	99.99
OXYCODONE-APAP 5/500 CAP	1	0.00	1084694	99.99
OXYCODONE-ASPIRIN 4.88-325 TA	1	0.00	1084695	99.99
OXYCODONE-IBUPROFEN 5-400 TAB	1	0.00	1084696	99.99
OXYCONTIN 10 MG TABLET	1	0.00	1084697	99.99
OXYCONTIN 10 MG TABLET SA	1	0.00	1084698	99.99
OXYCONTIN 20 MG TAB ER 12H	1	0.00	1084699	99.99
OXYCONTIN 20 MG TAB.SR 12H	1	0.00	1084700	99.99
OXYCONTIN 20 MG TABLET SA	1	0.00	1084701	99.99
OXYFAST 20 MG/ML SOLUTION	1	0.00	1084702	99.99
OXYMORPHONE HCL 10 MG TABLET	1	0.00	1084703	99.99
OXYMORPHONE HCL 5 MG TABLET	1	0.00	1084704	99.99
OXYMORPHONE HCL ER 10 MG TAB	1	0.00	1084705	99.99
OXYMORPHONE HCL ER 15 MG TAB	1	0.00	1084706	99.99
OXYTOCIN 10U/ML VIAL	1	0.00	1084707	99.99
PEDIATEX HC 17.5-1.67 LIQUID	1	0.00	1084708	99.99
PEDIATEX HC 20-1.67/5 LIQUID	1	0.00	1084709	99.99
PERCOCET 10/650 MG TABLET	1	0.00	1084710	99.99
PERCOCET 5/325 MG TABLET	1	0.00	1084711	99.99
PERCODAN 4.5-325MG TABLET	1	0.00	1084712	99.99
PERCOLONE 5MG TABLET	1	0.00	1084713	100.00
PHENAPHEN/CODEINE #3 CAP	1	0.00	1084714	100.00
PHENDACOF HC 120-6-2.5 SYRUP	1	0.00	1084715	100.00
POT GUAIACO-HYDROCODONE BIT 1	1	0.00	1084716	100.00
PROCET 7.5/325MG TABLET	1	0.00	1084717	100.00
PROPACET 100-650 TABLET	1	0.00	1084718	100.00
PROPOXYPHEN-APAP 100-500 MG T	1	0.00	1084719	100.00
PROPOXYPHENE HCL 65MG CAPSULE	1	0.00	1084720	100.00
PROPOXYPHENE-ACETAMINOPHEN TA	1	0.00	1084721	100.00
RELACON-HC NR LIQUID	1	0.00	1084722	100.00
REPREXAIN 2.5-200 MG TABLET	1	0.00	1084723	100.00
REZIRA 60-5MG/5ML SOLUTION	1	0.00	1084724	100.00
RHINACON DH 5-2.5MG/5 LIQUID	1	0.00	1084725	100.00
RINDAL HD 5-1.67-2/5 SYRUP	1	0.00	1084726	100.00
ROXICET 5 MG-500MG TABLET	1	0.00	1084727	100.00
ROXICODONE INTENSOL 20MG/ML	1	0.00	1084728	100.00
RYBIX ODT 50 MG TAB RAPDIS	1	0.00	1084729	100.00
RYZOLT 100 MG TBMP 24HR	1	0.00	1084730	100.00
RYZOLT ER 200 MG TABLET	1	0.00	1084731	100.00
SKYRIZI (2 SYRINGES) KIT 150M	1	0.00	1084732	100.00
SUBOXONE 2 MG-0.5 MG TABLET	1	0.00	1084733	100.00
SUBOXONE 2 MG-0.5MG TAB SUBL	1	0.00	1084734	100.00
SUBUTEX 8 MG TABLET	1	0.00	1084735	100.00
TALACEN 25-650MG TABLET	1	0.00	1084736	100.00
TL-HIST CD 4-7.5-10/5 LIQUID	1	0.00	1084737	100.00
TRAMADOL HCL ER 100 MG TBMP 2	1	0.00	1084738	100.00
TREZIX 356-30-16 CAPSULE	1	0.00	1084739	100.00

TREZIX CAPSULE	1	0.00	1084740	100.00
TUSS-PD SYRUP	1	0.00	1084741	100.00
TUSSADUR-HD 100-30-2.5 ELIXIR	1	0.00	1084742	100.00
TUSSAFED-HC SYRUP	1	0.00	1084743	100.00
TUSSEND 60-5-4MG TABLET	1	0.00	1084744	100.00
TUSSEND TABLET	1	0.00	1084745	100.00
TUSSI-ORGANIDIN NR 300-10MG/5	1	0.00	1084746	100.00
TUSSI-ORGANIDIN-S NR 100-10MG	1	0.00	1084747	100.00
TUZISTRA XR 14.7-2.8/5 SUS ER	1	0.00	1084748	100.00
TY-PAP W/CODEINE ELIXIR	1	0.00	1084749	100.00
TYLENOL-CODEINE NO.4 300MG-60	1	0.00	1084750	100.00
ULTRAM ER 300 MG TAB ER 24H	1	0.00	1084751	100.00
VANEX HD 5-1.67-2/5 SYRUP	1	0.00	1084752	100.00
VEGETABLE LAXATIVE POWDER	1	0.00	1084753	100.00
VEGETABLE LAXATIVE POWDER	1	0.00	1084754	100.00
VICODIN ES 7.5-750MG TABLET	1	0.00	1084755	100.00
VICODIN HP 10-660MG TABLET	1	0.00	1084756	100.00
VITUSSIN EXPECTORANT	1	0.00	1084757	100.00
WELLTUSS HC LIQUID	1	0.00	1084758	100.00
WYGESIC 65/650 TABLET	1	0.00	1084759	100.00
XODOL 5-300 TABLET	1	0.00	1084760	100.00
XODOL 7.5-300 7.5-300MG TABLE	1	0.00	1084761	100.00
Z-COF HC 8-3.5-2.5 SYRUP	1	0.00	1084762	100.00
Z-COF HCX 200-7.5/5 LIQUID	1	0.00	1084763	100.00
ZOHYDRO ER 10 MG CAP ER 12H	1	0.00	1084764	100.00
ZOLVIT 10 MG-300 MG/15 ML SOL	1	0.00	1084765	100.00
ZYDONE 5 MG-400MG TABLET	1	0.00	1084766	100.00
ZYDONE 7.5-400 MG TABLET	1	0.00	1084770	100.00
